# Comparison of keel bone traits, eggshell production, and physiological parameters between a hybrid layer and two low-performing chicken genotypes

**DOI:** 10.3389/fphys.2026.1782139

**Published:** 2026-03-13

**Authors:** Lisa Hildebrand, Saskia Neukirchen, Mareike Fellmin, Julia Mehlhorn, Stefanie Petow, Lars Schrader, Steffen Weigend, Beryl Eusemann-Keller

**Affiliations:** 1 Friedrich-Loeffler-Institut, Institute of Animal Welfare and Animal Husbandry, Celle, Germany; 2 Institute for Animal Science – Farm Animal Ethology, University of Bonn, Bonn, Germany; 3 Poultry Research Center, Bruno-Dürigen Institute, Rommerskirchen, Germany; 4 Institute for Anatomy I, Medical Faculty and University Hospital Düsseldorf, Heinrich Heine University Düsseldorf, Düsseldorf, Germany; 5 Chair of Animal Welfare, Ethology, Animal Hygiene and Animal Husbandry, Department of Veterinary Sciences, Faculty of Veterinary Medicine, Munich, Germany; 6 Friedrich-Loeffler-Institut, Institute of Farm Animal Genetics, Neustadt-Mariensee, Germany; 7 University of Goettingen, Center for Integrated Breeding Research, Göttingen, Lower Saxony, Germany; 8 Leipzig University, Faculty of Veterinary Medicine, Institute of Animal Hygiene and Veterinary Public Health, Leipzig, Germany

**Keywords:** animal welfare, estradiol (17ß-estradiol), Junglefowl, keel bone damage, keel bone fractures, laying hens, radiography

## Abstract

**Introduction:**

Keel bone damage is a severe animal welfare problem in laying hens. Although it is influenced by husbandry and diet, the selection for laying performance seems to play a key role.

**Materials and Methods:**

In order to learn more about the pathogenesis of keel bone damage, this study aims to characterize and compare keel bone health and potentially related traits in Lohmann Selected Leghorn (LSL, n = 12) and two low-performing genotypes that have not intensively been selected for laying performance: Junglefowl phenotype (JF, n = 14) and Sumatra (Su, n = 12). X-ray imaging, blood sampling, and ultrasonography were conducted at five different time points between the 16th and 72nd week of age. The X-ray images were evaluated for fractures, deviated keel bone area, radiographic density, length, and ossification of the keel bone. Blood samples were used to determine blood ionized calcium, as well as plasma total calcium, phosphate, and 17-β-estradiol. Laying activity and eggshell quality were assessed at group level. Ultrasonography was used to detect visible follicles and assess pectoral muscle thickness.

**Results:**

Keel bone fractures were detected in five out of twelve LSL hens, but in none of the two low-performing genotypes. The first egg was laid distinctly earlier in LSL than in JF and Su (18th vs. 24th and 31st week of age, respectively). Keel bone ossification was completed significantly later in Su than in LSL and JF, but there was no significant difference between the latter two. Visible follicles at the ovary were associated with significantly higher plasma calcium and 17-β-estradiol levels.

**Discussion:**

This study provides a deeper insight into keel bone health and related traits in LSL and low-performing chicken genotypes. Our findings indicate that the earlier onset of lay in LSL does not correspond with earlier keel bone maturation, which could increase later susceptibility for keel bone fractures.

## 1 Introduction

Skeletal integrity is an important animal welfare parameter in laying hens (Nielsen et al., 2023). The term “keel bone damage” summarizes two types of lesions of the keel bone: fractures and deviations. Fractures are defined as a break in the continuous structure of the bone cortex (Sheen et al., 2025). Keel bone deviations are defined as abnormalities in keel bone shape that are not caused by fractures (Casey-Trott et al., 2015). Keel bone fractures have negative effects on the affected animal and its economic performance: They lead to pain, reduced mobility and laying performance, and an increase in feed and water consumption (Nasr et al., 2012; Armstrong et al., 2020; Nasr et al., 2013). It has also been suggested that keel bone damage could affect the animal’s respiration as the responsible muscles attach to the keel bone (Riber et al., 2018). With prevalence rates ranging from 50% to 100%, both keel bone fractures and deviations are extremely common in laying hens in practice as well as under experimental conditions (Käppeli et al., 2011; Baur et al., 2020; Casey-Trott et al., 2015; Thøfner et al., 2021; Jung et al., 2019). Although housing and diet have an influence on bone health and the occurrence of keel bone fractures (Eusemann et al., 2018a; Wilkins et al., 2011; Toscano et al., 2018), other, hen-related factors (e.g., behavior, genetics, and performance) also play an important role in the pathogenesis of keel bone damage.

While keel bone deviations occur in chickens of both sexes and even other species such as quails (Hildebrand et al., 2024) and turkeys (North, 1939), keel bone fractures are closely linked to egg laying, with few to no fractures being found in roosters or pullets (Kittelsen et al., 2021). The relationship between egg laying and keel bone fractures has also been demonstrated by Eusemann et al. (Eusemann et al., 2020) who used deslorelin acetate implants to prevent hens from laying. This reduced the risk for developing keel bone fractures by 80%. Correspondingly, in some studies, layer lines with higher laying performance showed more frequent and more severe keel bone damage (Eusemann et al., 2018a; Habig et al., 2021a). However, within a layer line, when strong outliers were excluded, a strong negative correlation between individual laying performance of laying animals and bone quality was not observed (Jansen et al., 2020). Studies that target traditional breeds, crossbreeds and Red Junglefowl, one of the non-domesticated ancestor species of modern chicken, show that these animals seem to be less susceptible to fractures than hybrid layers (Jung et al., 2024; Pulcini et al., 2023). This indicates that even though keel bone damage may occur in all chicken genotypes, the selection for high laying performance could have intensified the issue. Possible underlying mechanisms are the constantly high calcium demand for the formation of the eggshell, the influence of reproductive hormones, especially 17-β-estradiol, on bone metabolism, and skeletal immaturity at onset of lay.

The main mineral component of bones is hydroxyapatite, which consists of calcium and phosphate (Whitehead and Fleming, 2000). During the laying phase, hens have an additional daily calcium demand of 2–3 g for the formation of the eggshell (Ajala et al., 2018). This calcium demand is only partly covered through dietary calcium. About 30%–40% of the calcium in the eggshell originates from the skeleton (Mueller et al., 1964; Farmer et al., 1986). As a highly dynamic calcium reservoir, female birds form a special type of bone during the laying phase. This medullary bone works as a labile calcium source (Whitehead, 1992; Bloom et al., 1958). Its formation is induced by the reproductive hormone 17-β-estradiol (Hiyama et al., 2012) which is secreted by the largest four hierachical and prehierachcial follicles on the ovary (as reviewed in Mans and Taylor, 2008). It has been suggested that besides the competitive calcium situation the shift of the bone metabolism towards the formation of medullary bone could lead to reduced bone stability (Wilson and Thorp, 1998; Whitehead and Fleming, 2000).

Bone stability is significantly linked to bone mineral density (Donkó et al., 2018). On X-ray images, radiographic density can be measured as a proxy for bone mineral density using a method established by Fleming et al. (2004). The authors showed that the *in vivo* determined radiographic density of the ulna and the humerus correlated with the *post mortem* measurements, which were taken after the bones were removed from the body. The method was later adapted for estimating keel bone density in laying hens *in vivo* (Eusemann et al., 2020). However, it has to be considered that the keel bone may be surrounded by more muscle tissue compared to the ulna and the humerus, which could influence the measured radiographic density. This may especially be of high importance when comparing different genotypes that differ in muscle distribution.

Another laying-related factor that may play a role in the development of keel bone fractures is the early onset of lay in modern laying hens (Gebhardt-Henrich and Fröhlich, 2015). As ossification proceeds from cranial to caudal and is not completed until between the 25th and the 40th week of age, depending on the genotype (Casey-Trott et al., 2017; Fawcett et al., 2020; Buckner et al., 1948), the caudal part is most likely not yet ossified when hybrid layers start laying at about 20 weeks of age (Thiele, 2012). It has been proposed that this negatively affects bone stability and, thus, increases the susceptibility for fractures (Toscano et al., 2020). This hypothesis is supported by the fact that the majority of fractures are found in the caudal third of the keel bone - the region that ossifies last (Kittelsen et al., 2021; Habig et al., 2021a; Pulcini et al., 2023).

Besides laying activity, the genetics of a hen strongly influence their bone quality and health (Warren, 1937; Dunn et al., 2007). Chicken genotypes can differ in various aspects, not only in terms of bone properties but also behavior as space usage, fearfulness, and spatial skills (Adler et al., 2024; Jansen et al., 2020; Nelson et al., 2020; Rentsch et al., 2023), all of which can influence the risk of keel bone fractures.

So far, only little is known about keel bone development and other possible bone-health-relevant traits in low-performing chicken genotypes that have not intensively been selected for laying performance. Thus, the aim of the current study was a systematic, longitudinal investigation and comparison of keel bone health, development, and associated parameters in a hybrid layer and two different low-performing genotypes, in order to learn more about the pathogenesis of keel bone damage. Thereby, we also explored the influence of individual body weight and pectoral muscle thickness on radiographic density, to assess the possible impact of morphological differences between and within the genotypes on this parameter. Based on previous studies, we hypothesized that keel bone fractures would occur more frequently in LSL than in the other two genotypes and, in addition, that the lower laying performance would be reflected in lower levels of reproductive hormones, total calcium, and phosphate in the blood plasma. Furthermore, we hypothesized that the two low-performing genotypes would reach sexual maturity later and that this would reflect in later completion of keel bone ossification.

## 2 Animals, materials, and methods

The animal experiment was approved by the State Agency for Nature, Environment and Consumer Protection of North Rhine-Westphalia (Reference number: 81-02.04.40.2023.VG039).

### 2.1 Animals and husbandry

In this trial, three different chicken genotypes were investigated: Lohmann Selected Leghorn (LSL), Junglefowl phenotype (JF), and Sumatra (Su). The Lohmann Selected Leghorn is a widely used white egg layer hybrid with a high laying performance of over 320 eggs/year (Preisinger, 2018). Junglefowl are the ancestor species of domestic chickens (Jain et al., 2024). They are relatively small and active, and typically lay their eggs in small clutches (Schmidt, 1999). Their annual laying performance in the wild is reported to amount to 10–15 eggs (Romanov and Weigend, 2001). The chickens that were investigated in this study showed physical and behavioral characteristics of Junglefowl. However, since the line investigated in this study originated from different non-commercial breeders, their genetic purity is not confirmed. Thus, they are referred to as Junglefowl phenotype. Sumatra represent ornamental fighting-type chickens that originate from feral domestic chickens on Indonesian islands and have not been selected for laying performance (Schmidt, 1999). According to the German association of fancy poultry breeders, the Bund Deutscher Rassegeflügelzüchter (BDRG), Sumatra can lay up to 130 eggs (Rassetafeln Huehner, 2024).

All experimental animals hatched and were kept at the Bruno-Dürigen Institute in Rommerskirchen. The hatching eggs of LSL were obtained from Lohmann Deutschland GmbH and Co. KG (Ankum, Germany). The hatching eggs for the low-performing genotypes were either produced on site (all JF, 9/14 Su) or acquired from a fancy poultry breeder that collaborated with the Bruno-Dürigen Institute (5/14 Su). They were then incubated in artificial brooders (Favorit-Olymp series, HEKA Brutgeräte GmbH and Co. KG, Rietberg, Germany). Pre-incubation was carried out at 37.8 °C and 53% humidity, hatching at 37.5 °C and 63%–80% humidity with an increase from day 19. From the first to the 18th day of incubation, the eggs were automatically tilted three times in 120 min. Due to low fertilization rates, a second batch of eggs was added after 2 weeks of brooding to achieve the requested group size. Thus, there was an age difference of 2 weeks between some of the animals. The animals hatched early to mid-April which corresponds to spring in North Rhine-Westphalia, Germany where they were kept. All animals of one genotype were kept together but separately from the other two genotypes. The final experimental groups were selected according to the phenotypic breed standards and contained 12 (LSL and Su) or 14 (JF) hens, which were each accompanied by two roosters. Between the beginning and end of the experiment, one Su and one JF hen dropped out due to death and two JF hens were removed from the experiment, because they were transferred to a different substudy. During the experiment, the number of roosters in each group was reduced to one due to death or aggression towards the animal caretakers. Furthermore, for breeding purposes, the remaining rooster in the JF group was replaced by one that did not take place in the experiment. The exact number of assessed animals per time point and investigation is given in the figures of the results section.

Housing conditions were similar for all three genotypes. For rearing, the animals were housed in 4.3 m^2^ indoor compartments that were equipped with brooder plates, infrared and radiant heaters, feeders, bell drinkers, and 2.5 cm × 4 cm rhomboid perches at 12, 30, 50, 70, and 90 cm of height with a length of 2 m each. From the 6th/8th week of age onward, brooder plates and heat lamps were only switched on at night and from the 8th/10th week of age onward, they were turned off completely. From the 3rd/5th week of age onward, the chickens had access to outdoor enclosures with a floor area between 5.9 and 7.4 m^2^. After 3 months, chickens were relocated to extensive outdoor enclosures (125–205 m^2^) with various natural and manmade enrichment elements and a 5.1 m^2^ chicken house. Each chicken house was equipped with a 2.8 m long wooden perch with a rectangular cross-section of approximately 4 cm × 6 cm. The perch was placed just above a dropping board at a height of about 90 cm. Nest size depended on the genotype. JF were offered three nests measuring 25 cm × 30 cm while LSL and Su were each provided with three or two 40 cm × 40 cm nests, respectively. All nests were placed at floor level. Feed and water were provided through 34 cm diameter bell drinkers and round feeders with a diameter of 40 cm for Su and LSL and 30 cm diameter for JF. Outdoor access was provided during daylight. A lighting program with 14 h of light was applied until the 35th/37th weeks of age. Afterward, lighting corresponded to the natural daylight that passed through two windows with a total area of 1.8 m^2^. The natural photoperiod ranged between 7.9 h in the 36th/38th week of age and 16.6 h in the 62nd/64th week of age.

LSL and Su received complete feed for chicks, pullets and laying hens produced by Deutsche Tiernahrung Cremer GmbH and Co. KG (Duesseldorf, Germany). Until 7 weeks of age, they were fed All-Mash A (11.4 MJ ME/kg, 18% CP, 1% Ca, 0.6% P), followed by All-mash R (11.4 MJ ME/kg, 14.5% CP, 0.9% Ca, 0.5% P). After the onset of lay, they received VoMiGo LAF (11.2 MJ ME/kg, 17% CP, 3.6% Ca, 0.5% P). Based on previous experience, JF were provided with special feed for ornamental poultry produced by Mischfutter Werke Mannheim (Mannheim, Germany). Until 6 weeks of age, they received ornamental poultry starter feed (11.6 MJ ME/kg, 26% CP, 1.3% Ca, 0.85% P). From then onward, they were fed pelleted feed for breeding and management of ornamental poultry (10.2 MJ ME/kg, 20% CP, 5% Ca, 0.75% P). Additionally, all animals were provided with grit and were occasionally fed with boiled eggs, fruit, vegetables, and grain mix.

### 2.2 Assessment of laying activity and egg quality

Laying activity was investigated at group level. The age at first egg and age at which 10% group laying performance was reached were recorded for each group. For the assessment of laying performance, eggs were counted for 1 year from when the respective group had reached 10% group laying performance onward.

For each group, egg quality was assessed during three 2-week sampling periods at the midpoints of the first, second, and last thirds of the first laying year, starting at 10% group laying performance. Each intact egg laid during the sampling period underwent the following measurements: First egg weight was measured with a KERN 440 precision scale (Kern GmbH, Großmaischeid, Germany). Subsequently, in order to determine eggshell breaking strength, the egg was placed then in the metal rack of the Fast-Egg-Shell-Tester (BRÖRING Technology GmbH, Lohne, Germany) with the pointed end in contact to the sensor plate. After activation, the egg was pressed against the sensor plate until the eggshell cracked. Thereby, the sensor plate measured the applied force in Newton. After the eggs were cracked open completely, the remaining egg liquid was drained from the eggshells and the shells were weighed on the KERN 440 precision scale without additional drying. Next, a small piece of eggshell was broken off from the equatorial region of the egg and its thickness was measured in a special micrometer screw with a rounded measuring surface (BRÖRING Technology GmbH, Lohne, Germany). The relative eggshell weight was determined by dividing the shell weight by the egg weight.

### 2.3 Sampling schedule for keel bone and blood parameters

Radiography, ultrasonography, and blood sampling took place at a total of five time points throughout rearing and laying period. The first three examinations took place in the 16th, 25th, and 33rd week of age. For these, animals of the two different age groups were examined 2 weeks apart to make sure that they were at the same age at the time of examination. This was especially relevant since ossification of the keel bone can take up to over 40 weeks of age in some genotypes (Buckner et al., 1949). The last two examinations were carried out at the same day for both age groups as the keel bone was supposed to be completely ossified. Thus, animals were examined in the 50th or 52nd week of age and in the 70th or 72nd week of age, respectively, depending on their hatching date. As part of the data collection, the animals were weighed using a hanging scale (Kern and Sohn GmbH, Balingen, Germany).

### 2.4 Radiography

For the radiographic examination of the keel bone, the animals were positioned in a rack as described by Hildebrand et al. (2024). Latero-lateral X-ray images were taken at 50 kV and 2.5 mA as described by Eusemann et al. (2018a) using the X-ray generator WDT Blueline 1040 HF (Wirtschaftsgenossenschaft Deutscher Tierärzte eG, Garbsen, Germany) and the digital flat panel detector Thales Piximum 2430 EZ wireless (Thales Electron Devices S.A., Velizy-Villacoublay, France) connected to a laptop (Latitude E5570, Dell Technologies Inc., Round Rock, United States). A home-built 2 cm × 17.8 cm aluminum step wedge with 18 steps between 0.5 mm and 4.75 mm thickness was placed on the detector plate for further analysis of the images, namely, for the assessment of the radiographic density.

**FIGURE 1:**
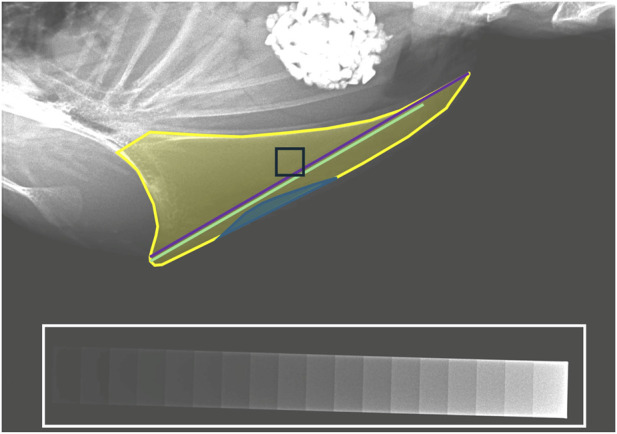
Measurement of continuous keel bone parameters. Total length (purple), length of the ossified part (light green), lateral surface area (yellow), and area of deviation (blue) were measured using the “length” and the “polygon” tool in ImageJ. An aluminum step wedge (white) was used to determine the radiographic density of the whole lateral surface area and a square placed in the middle of the keel bone (black).

The analysis of the X-ray images was performed with the software ImageJ (Version 1.48, Rasband, W., National Institute of Health, Bethesda, Maryland, United States) by one single observer. Intraobserver repeatability was assessed on basis of the images that were taken until the 50th/52nd woa and was 100% for the detection of fractures. For the assessment of fractures, the keel bone was subdivided into three equal sections from cranial to caudal adapted from Baur et al. (2020) using a customized ImageJ tool. For each third, the number of fresh fractures and calluses was scored separately on a scale with three levels: 0 = no fresh fracture/callus, 1 = one fresh fracture/callus, 2 = two or more fresh fractures/calluses. Fresh fractures were defined as radiolucent lines or gaps in the continuous structure of the keel bone and calluses as irregular spots with an increased radiodensity. For each image, the scaling was set according to the dimensions of the aluminum step wedge. Then, the total length, length of the ossified part, lateral surface area, and area of deviation were measured as shown in Figure 1 using the length and the polygon tool in ImageJ. As a proxy for bone mineral density, the radiographic density of the keel bone was measured as described by Eusemann et al. (2020). For each image, the gray values and the height of each step of the aluminum step wedge were used to generate an individual calibration curve with a Rodbard function (Rodbard, 1974). According to the calibration curve, the gray value of any selected part of the image could be calculated as *millimeters aluminum equivalent (mmAleq)*. The radiographic density was measured for both a) the whole keel bone, including fractures but excluding superimpositions, and b) a square in the middle of the keel bone. For the positioning of the square, a customized tool was created in ImageJ. The observer drew a straight line between the cranial and caudal tip of the keel bone (alternatively, the most caudal ossified point for not completely ossified keel bones). A square was then placed automatically in the middle of that line. Its side length was set at 1/12 of the length of the ossified part of the keel bone. If the square was not set in the middle of the keel bone by the tool (this was sometimes the case for deviated or highly curved keel bones), it was moved manually. To assess the severity of deviations, the proportion of deviated keel bone area (POD) was calculated as described in Eusemann et al. (2020), namely, by dividing the deviated part by the whole surface area and multiplying with 100. The degree of ossification was calculated by dividing the length of the ossified part by the total length of the keel bone.

### 2.5 Ultrasonography

Transcutaneous ultrasonography was used to observe the follicle status of the hens from 25 weeks of age onward using the ultrasound scanner CHISON SonoBook 6 Portable Ultrasound (CHISON Medical Technologies Co., Wuxi, Jiangsu, China) as described in Eusemann et al. (2018b). In short, the animals were positioned on their right side and the left leg was pulled slightly cranial so that the examiner could position a curved array transducer (Chison MC6-V 6.0 MHz Micro-Convex Probe, CHISON Medical Technologies Co., Wuxi, Jiangsu, China) caudal to the ribs. This unilateral procedure was adequate, as only the left ovary is fully developed in chickens. If dominant follicles (>10 mm) were detected, the animal was classified as F+ (animal with visible dominant follicles); otherwise, as F- (animal without visible dominant follicles). In three cases, no dominant follicle was found during the sonography, but an egg was visible during sonography or in the X-ray image, clearly indicating laying activity. Therefore, the follicle status was changed to F+. Since the first egg was laid in the 18th week of age, the follicle status for the 16th week of age was set to F- for all animals without conducting ultrasonography at this time point.

**FIGURE 2:**
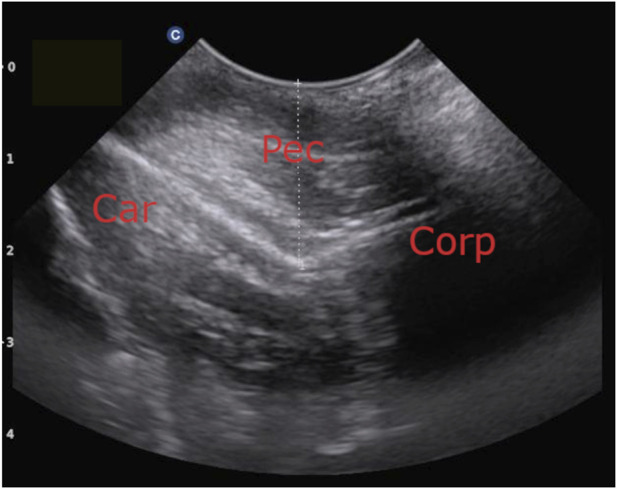
Measurement of pectoral muscle thickness using transcutaneous ultrasonography. The pectoral muscle thickness was measured from the skin to the bottom of the “V”-shape. Legend: Car = Carina Sterni, Corp = Corpus Sterni, Pec = Pectoral muscles.

In addition, from the 50th/52nd week of age onward, the thickness of the left pectoral muscles was measured in order to assess whether this parameter may influence radiographic density of the keel bone. The measurement was performed based on the approach presented in Garant et al. (2022) and adjusted as follows: Instead of using a linear transducer, the same curved array transducer was used as for the sonography of the follicles. Instead of using a plastic vernier caliper to determine the position of the transducer, it was placed cranial to the keel bone and then moved caudal until both corpus and carina of the keel bone became visible (Figure 2). Then, a screenshot was taken, and the distance from the skin to the bottom of the “V”-shape, where carina and corpus sterni meet, was measured.

### 2.6 Blood sampling

Blood sampling was always performed between 10:30 a.m. and 3 p.m. using peripheral venous catheter (Vasofix® Braunüle® G 22, B. Braun SE, Melsungen, Germany) and Lithium Heparin micro tubes (Sarstedt AG and Co. KG, Nuembrecht, Germany). Blood samples were taken from the ulnar vein and a maximum of 2.6 mL were taken from each animal per time point. From the 25th week of age onward, ionized calcium was measured directly after the sampling. For later analysis of the concentrations of 17-β-estradiol, total calcium, and phosphate, the samples were cooled down on crushed ice, centrifuged at 4,600 rpm and 4 °C–10 °C for 10 min with the Thermo Scientific™ Heraeus™ Fresco centrifuge (Thermo Fisher Scientific, Waltham, Massachusetts), transported on dry ice to the facilities of the Friedrich-Loeffler-Institut, and stored at −80 °C until further analysis.

### 2.7 Analysis of minerals and reproductive hormones

The content of ionized calcium in the blood was measured using a portable device (LAQUATwin-Ca-11C, Horiba Ltd., Kyoto, Japan). To that aim, directly after sampling, 300 µL of blood were taken from the tube using a pipette (Eppendorf® Reference 1000, Eppendorf SE, Hamburg, Germany) and placed onto the electrode.

The concentrations of total calcium and phosphate in the blood plasma were measured with an Indiko™ Clinical Chemistry Analyzer (Thermo Fisher Scientific, Waltham, Massachusetts). Commercial ELISA kits were used for the measurement of the plasma concentration of 17-β-estradiol according to manufacturer’s instructions (ABIN RE52041, IBL International GmbH, Hamburg, Germany). All measurements were performed in duplicate and repeated when the coefficient of variation exceeded 15% and standard deviation exceeded 10.

### 2.8 Statistical analysis

The data analysis was conducted using the software RStudio (Version 2024.09.0, Posit PBC, Boston, Massachusetts, United States). Only hens were included in the statistical analysis. Levene’s tests were used to assess homoscedasticity. Residuals were checked for normal distribution using Q-Q-plots, histograms, and scatter plots. If necessary, data were transformed in order to reach normal distribution. Heteroscedasticity was addressed by using robust standard errors (HC3) in the analysis of linear models or by modeling heterogenous variances using varIdent in linear mixed models (nlme package).

Results with p < 0.05 were considered statistically significant. Due to the exploratory nature of this study, results approaching significance (p < 0.1) were also reported and discussed as potential trends. For multiple comparisons without a significant difference, the smallest p-value observed is reported as (p > X).

#### 2.8.1 Laying performance and egg quality

The average laying performance per hen and year was calculated by dividing the number of eggs laid during the first year after the group had reached 10% laying performance by the number of hens. For LSL and Su, egg numbers were missing for seven and 4 days, respectively. Therefore, the available data were extrapolated to 365 days.

Before the analysis of the eggshell quality, implausible measurements due to technical problems and outliers (<Q1 - 1.5 IQR and > Q3 + 1.5 IQR) were excluded from the data set. For egg weight, eggshell weight, relative eggshell weight (log-transformed), eggshell thickness, and breaking strength, linear models with sampling period, genotype, and their interaction as fixed effects were built and an ANOVA was performed. Due to heteroscedasticity, robust standard errors (HC3) were used for the analysis of eggshell weight and thickness.

#### 2.8.2 Keel bone fractures

Due to the relatively small amount of data, the results on keel bone fractures were simplified into a binary variable: 0 = no fresh fracture or callus in any of the three keel bone sections, 1 = at least one fresh fracture or callus in any of the three keel bone sections. In 20 radiographs (4 from JF and 16 from Su), there were strong superimpositions of the leg and the caudal tip of the keel bone. As this could interfere with the detection of fractures, the respective images were excluded from statistical analysis. A logistic regression model with bias reduction (Firth-method, brglm package) was used to analyze the influence of age and genotype on the occurrence of keel bone fractures. LSL in the 16th week of age was chosen as reference group because it represented the earliest data collection time point and no fractures had occurred in this group at that time. Additionally, overall keel bone fracture prevalence was calculated using exact binomial 95% confidence intervals. Only animals that were investigated until the end of the study and did not show severe superimpositions of leg and keel bone were included in this analysis.

#### 2.8.3 Continuous keel bone parameters and body weight

To validate the measurement of the radiographic density in the square placed in the middle of the keel bone instead of measuring it for the whole keel bone, the correlation between the two values was investigated by drawing a scatter plot and calculating Pearson’s correlation coefficient.

For the analysis of body weight, keel bone length, radiographic density (measured in the square), POD (from the 25th week of age onward, square-root-transformed), and pectoral muscle thickness, linear mixed models with age and genotype and their interaction as fixed effects were built. To account for repeated measurements, the animal was included as a random effect. Additionally, for the analysis of radiographic density, the relative body weight of an animal within its genotype and age group was included as a fixed effect in the model to take the individual constitution into account. The relative body weight was calculated by dividing the body weight of an animal by the average body weight of the animals of the same genotype at the respective age. The p-values for the main effects and their interaction were obtained using the anova function. As the interaction between age and genotype was clearly not significant for pectoral muscle thickness and POD (p > 0.626), simplified models without the interaction term were fitted for these parameters. Tukey-Kramer tests were used for *post hoc* analysis. For the analysis of radiographic density, one outlier with an extreme residual and a deviating individual trend was excluded. Additionally, the average adult body weight was determined for all three genotypes by calculating the mean from the 33rd, 50th/52nd, and 70th/72 nd weeks of age.

#### 2.8.4 Ossification

Due to data distribution, ossification could not be analyzed with a linear mixed model. Instead, a survival analysis with complete ossification as the event of interest was performed and a Log-rank test was used to evaluate differences between the genotypes. As in our experience, the surrounding fat tissue at the caudal tip of the keel bone can sometimes mislead to the impression that a keel bone is not yet fully ossified, we defined complete keel bone ossification as a state of keel bone ossification >95%. When animals dropped out of the experiment or strong superimpositions interfered with the measurement of the keel bone ossification, the data of this animal was censored from that time point onward. For *post hoc* analysis, pairwise log-rank tests with Holm adjustment for multiple testing were used.

#### 2.8.5 Blood parameters

Due to the amount and distribution of our data, it was not possible to analyze the influence of age, follicle status, and genotype on the blood parameters in one model. Therefore, for each blood parameter two linear mixed models were built: one with follicle status, genotype, and their interaction as fixed effects and another one with age, genotype, and their interaction as fixed effects. Both included the animal as random effect to account for the repeated measurements. Since the proportions of F+ and F- animals differed between the genotypes, the interaction term was retained in the model, even if it was not significant, to allow comparisons at group level. For 17-β-estradiol, outliers (Q1 -1.5 IQR and Q3 +1.5 IQR) were removed from the data set before the analysis. Total plasma calcium and 17-β-estradiol values were log-transformed for normal distribution. The p-values for the main effects and their interaction were obtained using the anova () function and Tukey-Kramer tests were used for *post hoc* analysis.

#### 2.8.6 Relationship between pectoral muscle thickness and radiographic density of the keel bone

To assess the relationship between pectoral muscle thickness and radiographic density, a scatter plot was drawn and Pearson’s correlation coefficient determined a) for all hens and b) within the genotypes.

## 3 Results

### 3.1 Laying performance and egg quality

LSL hens were the first to lay their first egg and to reach 10% laying performance, followed by JF and then Su (Table 1). The average laying performance per hen during the first year after the group had reached 10% laying performance was distinctly higher in LSL (315 eggs/year) than in JF (83 eggs/year) and Su (92 eggs/year).

**TABLE 1:** Time of first egg and 10% laying performance.

Genotype	LSL	JF	Su
Age at first egg [week of age]	18	24	31
Age at 10% laying performance [week of age]	21	26	32

The interaction between genotype and sampling period significantly affected the egg weight (F_4,411_ = 6.79, *p* < 0.001), eggshell weight (F_4,386_ = 9.59, *p* < 0.001), relative eggshell weight (F_4,383_ = 7.24; *p* < 0.001), and eggshell breaking strength (F_4,415_ = 5.17, *p* < 0.001) (Table 2). Eggshell thickness was significantly influenced by genotype (F_2,402_ = 99.52, *p* < 0.001) and sampling period (F_2,402_ = 2.45, *p* < 0.001). Although the interaction effect only approached significance (*p* = 0.087), it was kept in the model as an interaction since it was significant for the other eggshell parameters. During all three sampling periods, LSL eggs were significantly heavier than Su and JF eggs (*p* < 0.001) with Su eggs being significantly heavier than JF eggs (*p* < 0.001). In LSL and Su, there was a significant increase in egg weight between the first and second sampling period (*p* < 0.001 and *p* = 0.002, respectively). In contrast, JF did not show significant changes in egg weight between the sampling periods (p > 0.628). Throughout all three sampling periods, eggshell weight was significantly higher in LSL than in the other two genotypes (*p* < 0.001) and higher in Su than in JF (*p* < 0.001). In LSL, eggshell weight significantly decreased between the second and third sampling period (*p* < 0.001). In the other two genotypes, eggshell weight did not significantly differ between the sampling periods (*p* > 0.991). Accordingly, relative eggshell weight significantly decreased between the first and the second and between the second and third sampling periods (*p* < 0.001) in LSL, while JF and Su did not show significant changes in relative eggshell weight between the sampling periods (*p* > 0.237). Consequently, during the first sampling period, relative eggshell weight was higher in LSL than in the other two genotypes (*p* < 0.001). In the second sampling period, relative eggshell weight was higher in LSL and JF than in Su (*p* < 0.001) without significant difference between LSL and JF (*p* = 0.484) and in the third sampling period, there were no significant differences between the genotypes (*p* > 0.179). During the first two sampling periods, eggshell breaking strength was significantly higher in LSL than in the other two genotypes (*p* < 0.001). Furthermore, there was a trend of Su eggshells having a higher breaking strength than JF eggshells (p = 0.075). In the third sampling period, eggshell breaking strength was significantly higher in LSL than in JF (*p* = 0.003) with Su in between (*p* > 0.121). In LSL, eggshell breaking strength decreased with age (1^st^ - 2^nd^ sampling period: *p* = 0.061, 2^nd^ - 3^rd^ sampling period: *p* < 0.001). Within JF and Su, eggshell breaking strength did not differ significantly between the sampling periods (*p* > 0.958). Eggshell thickness did not differ significantly between the sampling periods in any genotype. However, during all three sampling periods, LSL had significantly thicker eggshells than both low-performing genotypes (p < 0.016) and Su had significantly thicker eggshells than JF (p < 0.001).

**TABLE 2:** Least squares means (LSM) and standard errors (SE) for egg weight, eggshell weight, relative eggshell weight, eggshell breaking strength, and eggshell thickness for eggs from LSL, JF and Su in the 1^st^, 2^nd^ and 3^rd^ Sampling period (SP) for eggshell quality.

Genotype x Sampling period (SP)		Egg weight [g]	Eggshell weight [g]	Relative eggshell weight [%]	Eggshell breaking strength [N]	Eggshell thickness [mm]
LSL	SP1	59.18 ± 0.3^d^	9.47 ± 0.07^d^	15.98 ± 0.12^e^	57.76 ± 0.92^c^	0.3947 ± 0.0023^c^
	SP2	64.77 ± 0.33^e^	9.48 ± 0.09^d^	14.6 ± 0.12^d^	53.62 ± 1^c^	0.3891 ± 0.0024^c^
	SP3	64.39 ± 0.32^e^	8.79 ± 0.08^c^	13.6 ± 0.11^bc^	45.69 ± 0.98^b^	0.3863 ± 0.0038^c^
JF	SP1	32.62 ± 0.63^a^	4.79 ± 0.09^a^	14.42 ± 0.28^cd^	37.33 ± 1.94^a^	0.3147 ± 0.0064^a^
	SP2	33.94 ± 0.4^a^	4.77 ± 0.08^a^	14.18 ± 0.16^cd^	36.28 ± 1.23^a^	0.3014 ± 0.0037^a^
	SP3	34.31 ± 0.64^a^	4.74 ± 0.02^a^	14.09 ± 0.4^abcd^	37.08 ± 1.98^a^	0.3082 ± 0.0065^a^
Su	SP1	46.24 ± 0.64^b^	6.33 ± 0.14^b^	13.64 ± 0.23^abc^	42.84 ± 1.98^ab^	0.3455 ± 0.0048^b^
	SP2	49.49 ± 0.48^c^	6.38 ± 0.08^b^	12.94 ± 0.17^a^	41.95 ± 1.46^ab^	0.3436 ± 0.0034^b^
	SP3	49.96 ± 0.66^c^	6.5 ± 0.11^b^	12.92 ± 0.23^ab^	39.51 ± 1.98^ab^	0.3603 ± 0.0064^b^

^a-d^Within a column, no common superscripts indicate significant differences (Tukey-Kramer, p < 0.05).

### 3.2 Body weight, keel bone, and blood parameters

The least squares means and standard errors for body weight, continuous keel bone measures and blood parameters are provided in the Supplementary Material.

#### 3.2.1 Body weight

**FIGURE 3:**
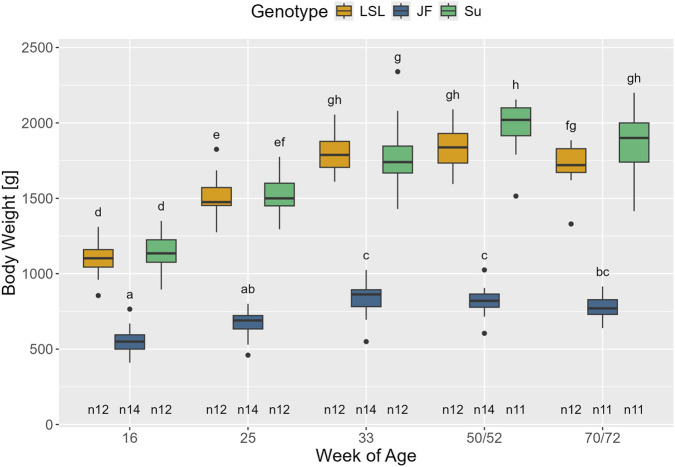
Body weight in relation to genotype and age. The x-axis represents age in weeks and the y-axis shows body weight in gram. Significant differences (Tukey-Kramer, p < 0.05) are indicated by no common subscripts. The number of animals can be viewed below the boxes of the boxplot (e.g., n12 indicates 12 animals).

The interaction between genotype and age significantly affected body weight (F_8,135_ = 23.68, *p* < 0.001) (Figure 3). In all three genotypes, body weight significantly increased between the 16th and 33rd week of age (*p* < 0.001). In LSL and JF, it then leveled off (*p* > 0.734), whereas Su showed a further significant increase in body weight between the 33rd and 50th/52nd week of age (*p* < 0.001). At all time points, JF had a significantly lower body weight than LSL and Su (*p* < 0.001) while there were no significant differences in body weight between Su and LSL (*p* > 0.524). The average adult body weight was 1782 ± 145 g for LSL, 812 ± 99 g for JF, and 1873 ± 228 g for Su.

#### 3.2.2 Keel bone fractures

**FIGURE 4:**
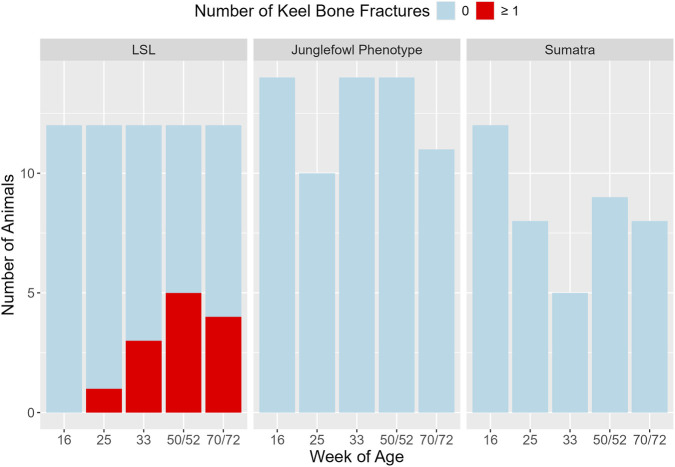
Number of hens with and without keel bone fractures at the respective time points in the different genotypes. The x-axis represents the age in weeks and the y-axis shows the number of animals with (red) and without (blue) keel bone fractures within the three different genotypes. Varying animal numbers in the low-performing genotypes are due to superimpositions of keel bone and tibiotarsus, which led to exclusion of the image from the analysis.

Keel bone fractures were only detected in LSL hens, not in JF or Su hens (Figure 4). Overall prevalence of keel bone fractures was 41.7% (95% exact binomial CI: 15.2–72.2) for LSL, 0% (95% exact binomial CI: 0–36.9) for JF, and 0% (95% exact binomial CI: 0–70.8) for Su.

All keel bone fractures were located in the caudal third of the keel bone. Only one fresh fracture was detected (LSL, 25th week of age). The remaining fractures were visible through callus formation. None of the genotype x age combinations showed a significant difference from the reference group (LSL in the 16th week of age). However, LSL in the 50th/52nd week of age showed a trend towards a significant difference (*p* = 0.071).

Overall, keel bone fractures observed in this study showed relatively little displacement and only minor to moderate callus formation.

#### 3.2.3 Keel bone deviations

No deviations were detected before the 25th week of age (Table 3). From the 25th week of age onward, the POD was not significantly affected by the interaction between genotype and age (*p* = 0.913). Therefore, a simplified model without interaction was chosen. There was a significant effect of genotype (F_2,35_ = 3.56, *p* = 0.039). The effect of age approached, but did not reach, the 5% significance level (F_3,106_ = 2.60, *p* = 0.056). POD was significantly higher in JF than in Su (*p* = 0.028) with LSL in between (*p* > 0.299).

**TABLE 3:** Proportion of deviated keel bone area (POD) in relation to genotype and age (arithmetic mean ± SD).

Genotype	Week of age				
	16	25	33	50/52	70/72
LSL	0.0% ± 0.00%	0.79% ± 0.93%	0.67% ± 0.42%	0.66% ± 0.59%	1.27% ± 1.44%
JF	0.0% ± 0.00%	1.37% ± 1.78%	1.24% ± 1.14%	1.33% ± 1.17%	1.91% ± 1.77%
Su	0.0% ± 0.00%	0.50% ± 0.93%	0.58% ± 0.77%	0.30% ± 0.81%	0.65% ± 0.69%

#### 3.2.4 Keel bone length

**FIGURE 5:**
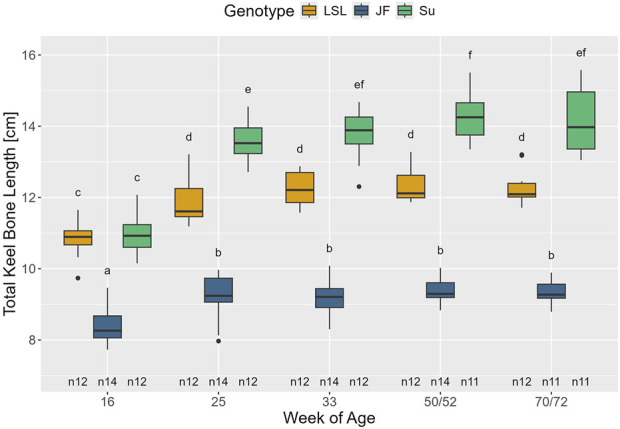
Total keel bone length in relation to genotype and age. The x-axis represents age in weeks and the y-axis shows total keel bone length in cm. The different genotypes are represented by different colors (see legend). Significant differences (Tukey-Kramer, p < 0.05) are indicated by different letters. The number of animals can be viewed below the boxes of the boxplot (e.g., n12 indicates 12 animals).

The interaction between age and genotype showed a significant influence on keel bone length (F_8,135_ = 17.23, *p* < 0.001) (Figure 5). Keel bone length increased in all three genotypes between the 16th and 25th week of age (*p* < 0.001). Furthermore, in Su, keel bone length was significantly higher in the 50th/52nd than in the 25th week of age (*p* = 0.029), which was not the case in the other two genotypes (*p* > 0.282). In the 16th week of age, LSL and Su had significantly longer keel bones than JF (*p* < 0.001), but there was no significant difference between the keel bone lengths of Su and LSL (*p* > 0.999). From the 25th week of age onward, Su had significantly longer keel bones than LSL and JF (*p* < 0.001) and LSL had significantly longer keel bones than JF (*p* < 0.001).

#### 3.2.5 Ossification

**FIGURE 6:**
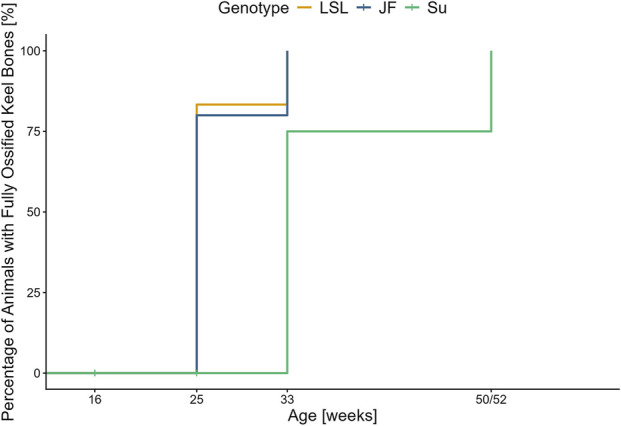
Percentage of hens with completed keel bone ossification (>95%) over time. The figure shows the percentage of animals with completed keel bone ossification (>95%) (y-axis) in relation to age in weeks (x-axis). The different genotypes are represented by different colors (see legend).

The genotypes significantly differed in terms of when animals reached complete keel bone ossification (*χ* ^2^ (Sheen et al., 2025) = 16.5, *p* < 0.001) (Figure 6). Ossification was completed significantly later in Su than in LSL (*χ* ^2^(1) = 12.64, *p* = 0.001) and JF (*χ* ^2^(1) = 10.93, *p* = 0.002). There was no significant difference between JF and LSL (*χ* ^2^(1) = 0.03, *p* = 0.844). In the 50th/52nd week of age, all keel bones were fully ossified.

#### 3.2.6 Radiographic density

The radiographic density of the whole keel bone strongly correlated with the radiographic density measured in the square placed in the middle of the keel bone (r (182) = 0.93, 95% CI [0.91, 0.95], p < 0.001). Thus, radiographic density measured in the square was used for further analysis.

**FIGURE 7:**
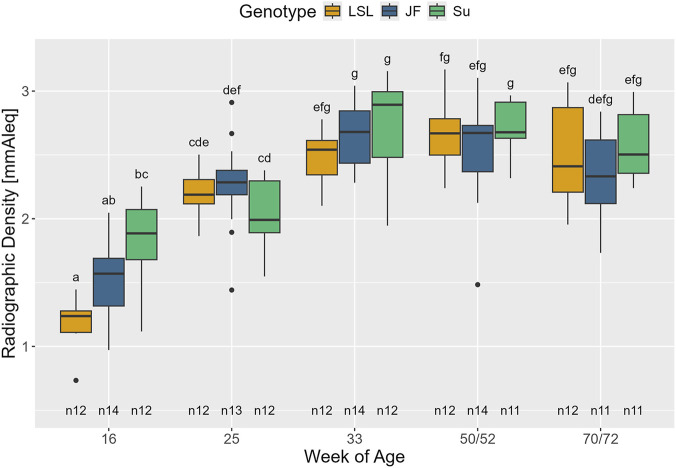
Radiographic density of the keel bone in relation to genotype and age. The x-axis represents age in weeks and the y-axis shows the radiographic density in millimeters aluminum equivalent. The radiographic density was measured in a square that was placed in the middle of the keel bone. The different genotypes are represented by different colors (see legend). Significant differences (Tukey-Kramer, p < 0.05) are indicated by different letters. The number of animals can be viewed below the boxes of the boxplot (e.g., n12 indicates 12 animals).

The interaction between age and genotype (F_8,133_ = 6.85, *p* < 0.001) significantly affected radiographic density (Figure 7). Furthermore, there was a significant positive effect of relative body weight (F_1,133_ = 33.05, *p* < 0.001, β = 1.23, SE = 0.21). In the 16th week of age, radiographic density was significantly higher in Su than in LSL (*p* = 0.004) with JF in between (*p* > 0.59). Thereafter, there were no significant differences between the genotypes at any time point (*p* > 0.68). In all three genotypes, radiographic density initially increased with age and then leveled off. There were significant increases in the radiographic density in LSL between the 16th and 25th and between the 25th and 50th/52nd weeks of age (*p* < 0.001 and *p* = 0.001, respectively). In JF, significant increases were observed between the 16th and 25th and the 25th and 33rd weeks of age (*p* < 0.001 and *p* = 0.014, respectively). Su showed a significant increase in radiographic density between the 25th and 33rd weeks of age (*p* < 0.001).

#### 3.2.7 Pectoral muscle thickness

**FIGURE 8:**
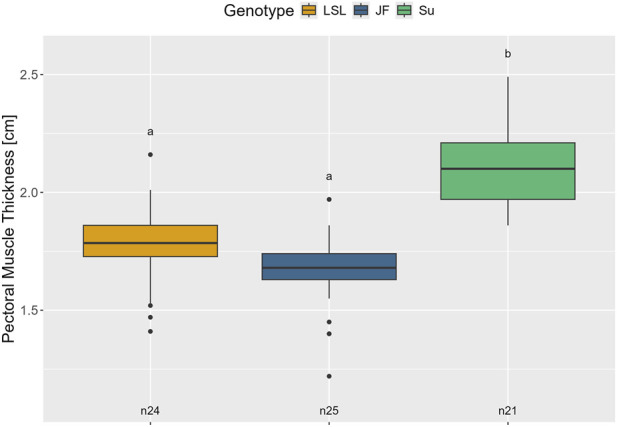
Pectoral muscle thickness in the different genotypes. The y-axis represents pectoral muscle thickness in cm. The different genotypes are represented by different colors (see legend). Significant differences (Tukey-Kramer, p < 0.05) are indicated by different letters. The number of animals can be viewed below the boxes of the boxplot (e.g., n24 indicates 24 measurements).

No significant interaction effect between genotype and age was found on pectoral muscle thickness (*p* = 0.627). Therefore, a simplified model without interaction was chosen. Pectoral muscle thickness was significantly influenced by genotype (F_2,34_ = 24.15, *p* < 0.001) but not by age (*p* = 0.138). As shown in Figure 8, Su had significantly thicker pectoral muscles than LSL and JF (*p* < 0.001) while pectoral muscle thickness did not significantly differ between LSL and JF (*p* = 0.218).

#### 3.2.8 Relationship between pectoral muscle thickness and radiographic density

**FIGURE 9:**
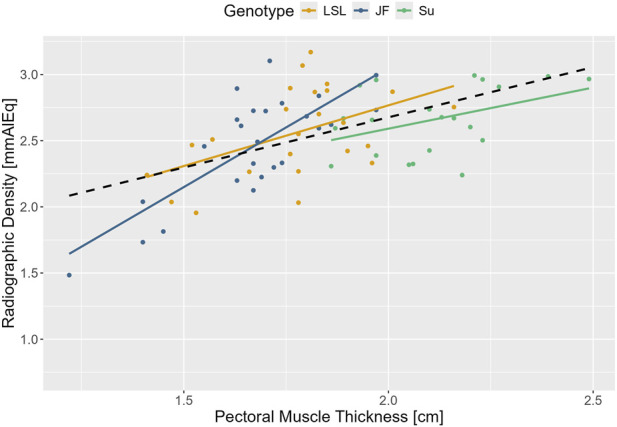
Correlation between pectoral muscle thickness and radiographic density. Pectoral muscle thickness is displayed in cm on the x- and radiographic density in millimeters aluminum equivalent on the y-axis. The different genotypes are represented by different colors (see legend). Regression lines are drawn to illustrate the correlation between the two parameters within the genotypes. The regression line across all animals is shown as a black dashed line.

There was a significant positive correlation between radiographic density and pectoral muscle thickness in the 50th/52nd and 70th/72nd weeks of age for all animals combined (r(69) = 0.55, 95% CI [0.36, 0.69], *p* < 0.001) and within the three genotypes: LSL (r(22) = 0.49, 95% CI [0.1, 0.74], *p* = 0.016), JF (r(23) = 0.77, 95% CI [0.55, 0.9], *p* < 0.001), and Su (r(20) = 0.42, 95% CI [0.00, 0.72], *p* = 0.0495) (Figure 9).

#### 3.2.9 Concentrations of plasma calcium, plasma phosphate, and blood ionized calcium

**FIGURE 10:**
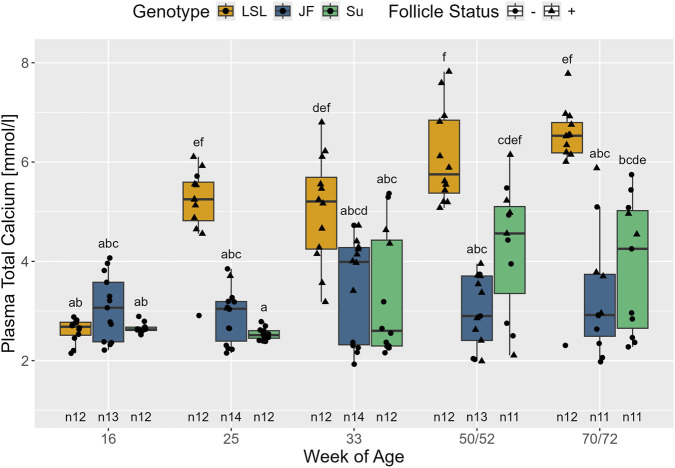
Plasma total calcium concentration in relation to age, genotype, and follicle status. The x-axis represents the age in weeks and the y-axis shows plasma calcium in mmol/l. The different genotypes are represented by different colors and point shapes indicate follicle status of the individual hen (see legend). Significant differences (Tukey-Kramer, p < 0.05) are indicated by different letters. The number of animals can be viewed below the boxes of the boxplot (e.g., n12 indicates 12 animals).

Both, the interaction between follicle status and genotype (F_2,142_ = 12.32, *p* < 0.001) and the interaction between age and genotype (F_8,133_ = 26.1, *p* < 0.001) significantly affected plasma calcium concentration (Figure 10). In all three genotypes, total calcium levels were significantly higher in F+ animals, i.e., in animals with dominant follicles on the ovary (*p* < 0.001). Within the F+ hens, LSL had significantly higher total calcium plasma levels than JF (*p* < 0.001) with Su in between (*p* > 0.125). Regarding the F- animals, there were no significant differences between the genotypes (*p* > 0.908). Across F+ and F- hens, plasma calcium significantly increased between the 16th and 25th week of age in LSL, (*p* < 0.001), but did not show significant changes thereafter (*p* > 0.363). In Su, plasma calcium levels across F+ and F- hens were significantly higher in the 50th/52nd week of age than in the 16th and 25th week of age (p = 0.008 and p = 0.002, respectively). Furthermore, they were significantly higher in the 70/72nd week of age than in the 25th week of age (*p* = 0.04). JF did not show significant changes in plasma calcium throughout the investigation (*p* > 0.971). From the 25th week of age onward, there was a consistent trend (*p* < 0.1) of LSL having higher calcium levels than Su and JF. However, the differences were only significant in the 25th week of age (p < 0.001) for both genotypes, in the 33rd week of age for Su (p = 0.037), and in the 50th/52nd and 70th/72nd weeks of age for JF (p < 0.001 and p = 0.002, respectively).

**FIGURE 11:**
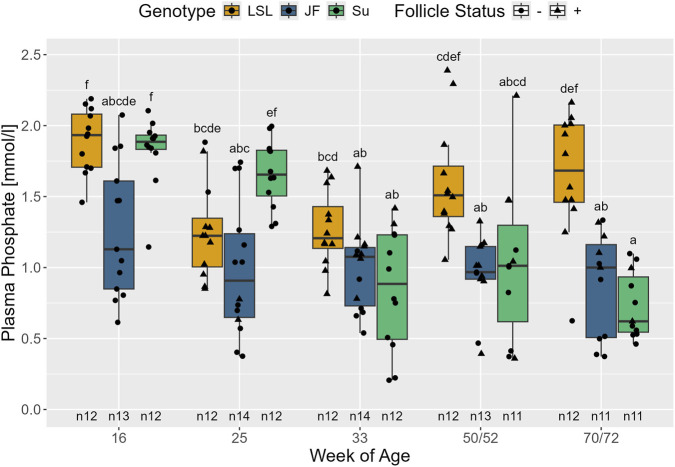
Plasma phosphate concentration in relation to age, genotype, and follicle status. The x-axis represents the age in weeks and the y-axis shows plasma phosphate in mmol/l. The different genotypes are represented by different colors and point shapes indicate follicle status of the individual hen (see legend). Significant differences (Tukey-Kramer, p < 0.05) are indicated by different letters. The number of animals can be viewed below the boxes of the boxplot (e.g., n12 indicates 12 animals).

Plasma phosphate concentration was also influenced by both: the interaction between follicle status and genotype (F_2,142_ = 3.64, *p* < 0.001) and the interaction between age and genotype (F_8,133_ = 9.56, *p* < 0.001) (Figure 11). LSL with negative follicle status had significantly higher plasma phosphate levels than LSL with positive follicle status (p = 0.037). However, in JF and Su, no significant differences were observed between F+ and F- animals (p > 0.996). JF did not show significant changes in plasma phosphate concentration with age (*p* > 0.897). LSL and Su had significantly higher plasma phosphate levels than JF in the 16th or 16th and 25th weeks of age, respectively (LSL: *p* = 0.013; Su: *p* = 0.046 and *p* = 0.002, respectively). Between the 25th and 33rd weeks of age, plasma phosphate in Su significantly decreased towards the level observed in JF (*p* < 0.001) and then leveled off (*p* > 0.94). Likewise, the plasma phosphate concentration of LSL was significantly lower in the 25th than in the 16th week of age (*p* < 0.001). Moreover, LSL had significantly higher plasma phosphate concentrations than JF (p = 0.003) in the 50th/52nd week of age and significantly higher plasma phosphate levels than both low-performing genotypes in the 70th/72nd week of age (JF: *p* = 0.006; Su: p < 0.001).

**FIGURE 12:**
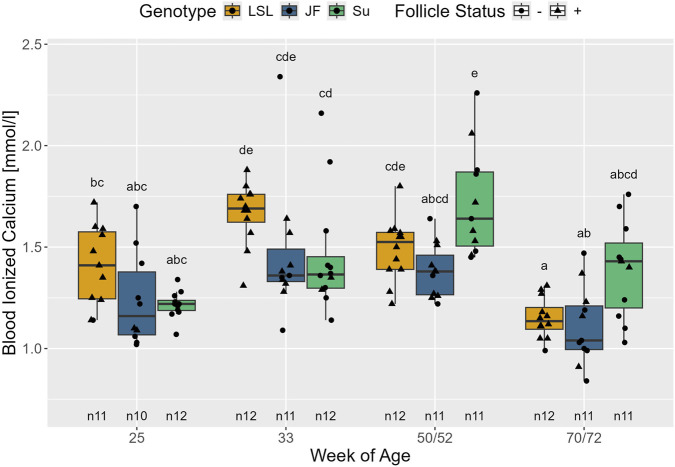
Blood ionized calcium in relation to age, genotype, and follicle status. The x-axis represents the age in weeks and the y-axis shows blood ionized calcium in mmol/l. The different genotypes are represented by different colors and point shapes indicate follicle status of the individual hen (see legend). Significant differences (Tukey-Kramer, p < 0.05) are indicated by different letters. The number of animals can be viewed below the boxes of the boxplot (e.g., n11 indicates 11 animals).

Blood concentration of ionized calcium was neither influenced by follicle status nor by the interaction between follicle status and genotype (*p* > 0.143). However, it was influenced by the interaction between age and genotype (F_6,89_ = 6.37, *p* < 0.001). In LSL, blood ionized calcium was significantly higher in the 33rd week of age than in the 25th week of age (*p* = 0.046) (Figure 12). In the 70th/72nd week of age, ionized blood calcium was significantly lower than at all other time points (25th week of age: *p* = 0.041; 33rd week of age and 50th/52nd week of age: *p* < 0.001). Likewise, in JF, blood ionized calcium levels were significantly lower in the 70th/72nd week of age than in the 33rd week of age (*p* = 0.001), but no significant differences were observed between the other time points (*p* > 0.083). In the 50th/52nd week of age, Su had significantly higher blood ionized calcium levels than at all other time points (25th week of age: *p* < 0.001; 33rd week of age: *p* = 0.029; 70th/72nd week of age: *p* = 0.003). A significant difference between the genotypes was only observed in the 50th/52nd week of age, with Su having significantly higher ionized calcium levels than JF (*p* = 0.010) with LSL in between (*p* > 0.230).

#### 3.2.10 Plasma levels of 17-β-estradiol

**FIGURE 13:**
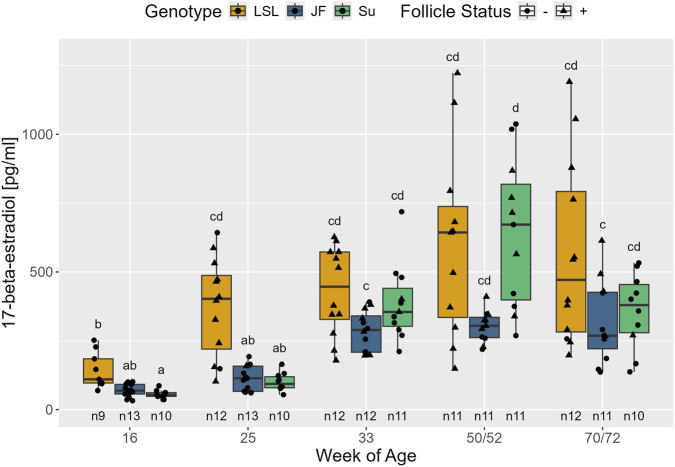
17-β-estradiol plasma levels in relation to age, genotype, and follicle status. The x-axis represents the age in weeks and the y-axis shows the concentration of 17-β-estradiol in pg/mL. The different genotypes are represented by different colors and point shapes indicate follicle status of the individual hen (see legend). Significant differences (Tukey-Kramer, p < 0.05) are indicated by different letters. The number of animals can be viewed below the boxes of the boxplot (e.g., n10 indicates 10 animals).

Plasma 17-β-estradiol was significantly influenced by genotype (F_2,35_ = 11.3, *p* < 0.001) and follicle status (F_1,127_ = 85.94, *p* < 0.001). Furthermore, the second model showed a significant influence of the interaction between genotype and age (F_8,119_ = 5.64, *p* < 0.001) (Figure 13). In all three genotypes, F+ animals had significantly higher 17-β-estradiol levels than F- animals (p < 0.001). Among the hens with negative follicle status, no significant difference was observed between the genotypes (p > 0.359). In F+ hens, LSL had significantly higher 17-β-estradiol levels than JF (p = 0.040) and Su showed a trend of having higher 17-β-estradiol levels than JF (p = 0.062), but there was no significant difference between Su and LSL (p = 0.968). Plasma 17-β-estradiol significantly increased between the 16th and 25th week of age in LSL (*p* < 0.001) and between the 25th and 33rd week of age in JF and Su (*p* > 0.001). In the 16th week of age, 17-β-estradiol levels were significantly higher in LSL than in Su (*p* = 0.015) and tended to be higher in LSL than in JF (*p* = 0.095). In the 25th week of age, LSL had significantly higher 17-β-estradiol levels than both low-performing genotypes (*p* < 0.001). From 33 weeks of age onward, there were no significant differences in plasma 17-β-estradiol between the genotypes (*p* > 0.216).

## 4 Discussion

### 4.1 Laying activity, egg quality, and body weight

The age at first egg differed between the genotypes. LSL laid their first egg in the 18th week of age, JF in the 24th week of age, and Su in the 31st week of age. For LSL and JF, this is consistent with the information from the literature (Preisinger, 2018; Schütz et al., 2002). As expected, laying performance was higher in LSL (315 eggs/year) than in JF (83 eggs/year) and Su (92 eggs/year). The laying performance of JF strongly deviates from the expected 10 to 15 eggs per year that are reported for Red Junglefowl living in the wild (Romanov and Weigend, 2001). This aligns with the initial assumption that the animals may not have been purebred of the wild genotype. However, laying performance was still low and also in line with the egg number documented for Red Junglefowl kept in captivity (Schütz et al., 2002).

LSL did not only lay more eggs than the other two genotypes. Their eggs were also significantly heavier and had stronger, heavier, and thicker eggshells, especially in the first and second sampling periods. While eggshell weight and breaking strength were sustained in low-performing genotypes, they decreased in LSL over time. While lower egg and eggshell weight in JF can be explained by their smaller body size, body weight was relatively similar between Su and LSL. Differences in eggshell quality between traditional breeds and hybrids have also been found by Lordelo et al. (2020), in whose study traditional Portuguese breeds had lower relative eggshell weights than hybrid hens, and Candelotto et al. (2017) who observed that the eggs of Bovans Brown hens had thicker eggshells than the ones laid by experimental brown layers, that descended from a line that had not been selected for specific breeding goal for the past years. Contrary to this, Hocking et al. (2003) did not see differences in eggshell breaking strength between commercial lines and traditional breeds. However, this investigation of eggshell quality took place in the 55th week of age, when eggshell quality measures already converged in our study. Decreases in eggshell quality over time are common in hybrid layers and have been reported and investigated by several researchers as reviewed in Roberts (2004). The findings suggest that LSL hens have a higher calcium demand per egg than JF and Su. In contrast to the two low-performing genotypes, LSL were not able to sustain eggshell quality. This underlines that producing large egg numbers with high eggshell quality occurs at the limits of physiological performance.

In all three genotypes, body weight significantly increased between the 16th and 33rd week of age. In LSL and JF, it then leveled off, while in Su, there was a further increase towards the 50th/52nd week of age. This increase could indicate that body growth takes longer in Su than in the other two genotypes, but could also be due to seasonal changes in body condition between winter and spring. Throughout the study, JF were significantly lighter than LSL and Su. These differences in body weight should be kept in mind when comparing the genotypes in terms of bone properties. Within a genotype, body weight positively correlates with several bone traits, such as bone mineral density (Kolakshyapati et al., 2019; Habig et al., 2021a).

### 4.2 Prevalence and severity of keel bone damage

In this study, no fractures were found in JF, Su, or roosters of any genotype (data not presented). Overall fracture prevalence was 41.7% (5/12) in LSL hens and 0% in JF and Su. However, the results do not necessarily indicate that JF and Su are free of keel bone fractures, as confidence intervals are relatively wide due to the small number of animals examined. Still, our findings are consistent with other studies that found fewer fractures in traditional breeds and crossbreeds with a medium laying performance compared to hybrid layers (Pulcini et al., 2023; Jung et al., 2024). In addition, the results are in range with other studies that found 10% fracture prevalence in Red Junglefowl (Kittelsen et al., 2021) and 61.1% in high-performing white layer lines (Eusemann et al., 2018a). Taken together, these findings indicate that genotypes that have not intensively been selected for laying performance are less susceptible to keel bone fractures. Still, fracture prevalence in LSL in this study was rather low compared to prevalence rates reported from commercial flocks (Thøfner et al., 2021). This could indicate that the housing conditions had a positive influence on keel bone health. It has to be taken into consideration that the results are limited due to the small number of animals examined in this study. In addition, some images from the low-performing genotypes had to be excluded from the data set as they could not be evaluated for fractures due to superimpositions of the caudal keel bone tip and the leg of the animal. Furthermore, it is possible that fractures occurred and healed between our radiographic examinations, as bone healing only takes about four to 6 weeks in birds (Dascălu et al., 2013). This could particularly be the case for fractures without displacement of the caudal tip of the keel bone. Regarding the fresh fracture in the 25th week of age, it should be noted that fracture morphology is not always a reliable indicator of the actual fracture age, as some fractures may not show the expected healing processes (Thøfner et al., 2020). All keel bone fractures were found in the caudal third of the keel bone. This is in accordance with other studies that reported that the majority of fractures are found in the caudal third of the keel bone (Habig et al., 2021a; Pulcini et al., 2023; Kittelsen et al., 2021).

No keel bone deviations were detected in the 16th week of age. This matches the observations by Habig et al. (2021a), who found only a small number of deviations at 15 weeks of age. In contrast, an older study (Warren, 1937) reports that the animals who developed deviations did so during the first 20 weeks of age. From 25 weeks of age onward, small deviations were present in all three genotypes. Even though POD appeared to slightly increase toward the 70th/72nd week of age, the effect of age was not statistically significant and deviations remained relatively mild (max. 5.37%). POD was slightly but significantly higher in JF than in Su with LSL in between. In a study conducted by Eusemann et al. (2018a), POD in floor-housed hens reached slightly higher values (max. 9.52%) but, likewise, did not increase significantly over time, while POD in cage-housed hens increased to higher POD values (max. 36.23%) throughout the laying period. Thus, our results support the hypothesis that physical activity as promoted through the housing conditions in our study may help to reduce the aggravation of deviations over time. Contrary to our results, Kittelsen et al. (2021) did not see keel bone deviations in JF. A possible explanation is that the relatively mild deviations were not detected, since palpation was used, which can lead to false-negative results for minor deviations (Casey-Trott et al., 2015). The fact that deviations were visible in all three genotypes emphasizes that deviations are most likely not related to egg-laying, but have a different etiology and pathogenesis than fractures. As the differences between the genotypes as well as the overall POD values were relatively small, the biological relevance has to be questioned. In addition, it has to be considered that radiography may not be the most suitable method to detect and investigate deviations, as it only provides two-dimensional images, which could lead to decreased sensitivity and specificity, especially for small deviations (Tracy et al., 2019; Hildebrand et al., 2024). So far, little is known about the impact of keel bone deviations on animal welfare. Therefore, more research is necessary to find out how keel bone deviations of different severities affect the animals.

### 4.3 Keel bone length and ossification

The genotypes significantly differed in keel bone length. Su had the longest keel bones, followed by LSL and JF. This underlines that although there is no significant difference in body weight between LSL and Su, they differ in body structure. As JF are generally lighter and smaller in size than the other two genotypes, it was expected that their keel bones would be shorter. Within the genotypes, keel bone length significantly increased between the 16th and 25th weeks of age. This increase was more pronounced in Su than in JF and LSL. These early changes in keel bone length most likely reflect keel bone growth. Additionally, in Su, keel bone length significantly differed between the 25th and 50th/52nd weeks of age. Since no significant difference was observed between the 25th and 72nd week of age, this was most likely due to measurement variations.

The results indicate that keel bone growth is completed later in Su than in the other two genotypes, which is in agreement with the findings for body weight. Likewise, ossification was completed significantly earlier in JF and LSL than in Su. In the 25th week of age, over 75% of the keel bones in JF and LSL were fully ossified, while keel bone ossification was not completed in any of the Su hens. At the following data collection, in the 33rd week of age, keel bone ossification was completed in all LSL and JF as well as in approximately 75% of the Su hens. The age at complete keel bone ossification in LSL and JF is consistent with the findings by Gretarsson et al. (2024) in Dekalb White, while the ossification in Su is completed at a later age, similar to what Buckner et al. (1948) found in New Hampshire chicken.

### 4.4 Radiographic density and pectoral muscle thickness

Our results show that the radiographic density measured in a square placed in the middle of the keel bone strongly correlates with the radiographic density of the whole keel bone. Besides saving time in the evaluation of the X-ray images, it is a convenient and consistent way to circumvent superimpositions and fractures, which interfere with the measurement of radiographic density.

In all three genotypes, radiographic density initially increased during the first half of the study period and then plateaued. This is similar to the pattern observed by Eusemann et al. (202). In the 16th week of age, radiographic density was significantly lower in LSL than in Su with JF in between. Afterward, there were no significant differences between the genotypes. Contrary to this, in Hocking et al. (2003), keel bone radiographic density at 55 weeks of age was slightly but significantly higher in traditional breeds than in commercial lines. However, the study investigated different breeds and measured radiographic density post-mortem after the extraction of the keel bones from the animals. The early differences in radiographic density in the 16th week of age could reflect differences in early keel bone mineralization between the genotypes. From the 25th week of age onward, the results indicate that the increase in radiographic density, and therefore presumably keel bone mineralization, proceeds approximately equally in all three genotypes. However, it has to be considered that *in vivo* radiographic density is most likely influenced by the tissue that covers the keel bone. A positive correlation between the radiographic density of the keel bone and the thickness of the left pectoral muscles was observed. This correlation was especially strong for JF. There are different possible explanations for this observation: the influence of the pectoral muscles on the measured grey value, the correlation between muscle mass and bone mineral density (Qin and Jiao, 2022) or a combination of both. Likewise, the significant effect of relative body weight on radiographic density could also be explained by either more surrounding tissue or the positive correlation between body weight and bone properties (Kolakshyapati et al., 2019). Further studies are necessary to investigate the correlation between keel bone radiographic density and actual keel bone mineral density and the influence of the surrounding (muscle-)tissue. Until then, radiographic density should be interpreted with caution as proxy for bone mineral density.

Regarding the pectoral muscle thickness, we did not see any significant effect of age. This outcome was expected as pectoral muscle thickness was only investigated at the last two examination dates, i.e., in the 50th/52nd and 70th/72nd weeks of age, and housing and possibility for exercise did not change between the two sampling dates. It matches the observations of Garant et al. (2022) who only saw changes in pectoral muscle thickness in fully immobilized white-feathered birds. There were significant differences in terms of pectoral muscle thickness between the genotypes. Su had significantly thicker pectoral muscles than LSL and JF, but there were no significant differences between the latter two. Su and JF seem to have a relatively higher pectoral muscle thickness in relation to body weight than LSL. For JF, this matches the observations in Endo et al. (2022) that showed that they had heavier pectoral muscles relative to body weight than other chicken genotypes. As described earlier, muscle mass positively correlates with bone mineral density (Qin and Jiao, 2022). In addition, pronounced pectoral muscles could help absorb the impact in case of collisions and protect the keel bone. As Toscano et al. (2018) showed, the risk for more severe keel bone fractures decreases with increasing breast muscle mass.

### 4.5 Blood and blood plasma minerals

Plasma total calcium was strongly linked to laying activity. In all three genotypes, F+ animals had higher plasma total calcium levels than F- animals. When analyzed in relation to age, plasma calcium increased in LSL and Su around onset of lay, while JF did not show significant changes throughout the study. The findings are supported by other studies that observed significantly higher plasma total calcium levels in hens during the laying phase than in pullets (Wei et al., 2021; Habig et al., 2021b; Parsons and Combs, 1981; Hanlon et al., 2022). In contrast to LSL, the hens of the low-performing genotypes laid less continuously after onset of lay and, thus, it is not surprising that they did not show such a clear effect with age. Additionally, differences between the genotypes were observed within the F+ animals: LSL had significantly higher plasma total calcium than JF with Su in between. These differences align with the findings of Grunder et al. (1980a), who showed that hens selected for thicker eggshells had higher serum calcium and vitellogenin, a calcium-binding protein, levels than hens with thin eggshells.

Plasma phosphate was significantly influenced by the interaction between age and genotype. While JF did not show any significant changes over time, plasma phosphate levels in LSL and Su decreased after the 16th or 25th week of age, respectively. In the 70th/72nd week of age the plasma phosphate was significantly higher in LSL than in the other two genotypes. Consistent with the findings in LSL and Su, other studies also reported a decrease in plasma phosphate at the start of the laying period (Wei et al., 2021; Qasir et al., 2025). However, in Habig et al. (2021b) an increase in phosphate between the 17th and 25th week of age was observed. After 25 weeks of age, the authors did not observe significant changes in plasma phosphate. LSL having higher phosphate plasma levels than the other two genotypes in the 70th/72nd week of age could be interpreted as a sign of increased bone resorption. Nevertheless, differences in plasma phosphate should be considered with caution, as they could also be due to changes in additional feed or diurnal variations (Habig et al., 2021b; Qasir et al., 2025). When investigating plasma phosphate in relation to follicle status, LSL with negative follicle status appeared to have higher plasma phosphate levels than F + LSL. However, it has to be taken into consideration that since LSL continuously laid eggs after the onset of lay, there is a strong association between age and follicle status. Nearly all LSL with negative follicle status were detected in the 16th week of age. Since there were no visible differences between the F+ and F- animals of the other genotypes, the observed difference is most likely due to the correlation with age and not caused by differences in mineral metabolism between laying and non-laying hens.

Blood ionized calcium was significantly affected by the interaction between age and genotype. However, no constant patterns could be observed. There was no significant effect of follicle status. Similar to these results, Habig et al. (2021b) did not see a constant trend regarding the effect of layer line on blood ionized calcium either. It has to be considered that in both pullets and laying hens, the blood content of ionized calcium varies during the day (Habig et al., 2021b; Parsons and Combs, 1981; Aysel, 1997) and can be influenced by blood pH (Sadiq et al., 2025; Wang et al., 2002) and contact of the blood sample to the ambient air (Pighi et al., 2024). As the timeframe for blood sampling was relatively wide (10:30 a.m. to 3 p.m.) and the method used to measure ionized calcium inevitably led to contact with ambient air and did not correct for pH differences, the results may be influenced by these factors and have to be interpreted with care. Overall, the higher levels of calcium and phosphorus in the feed for JF seemed not to result in higher blood and plasma concentrations.

Blood phosphate and calcium homeostasis is regulated by different hormones, such as parathyroid hormone (PTH) and calcitriol. For a better understanding and interpretation, measurements of these hormones could be incorporated into follow-up studies to provide additional context.

### 4.6 Plasma levels of 17-β-estradiol

Plasma 17-β-estradiol was influenced by laying activity. In all three genotypes, F+ hens showed higher 17-β-estradiol levels than F- hens. In addition, 17-β-estradiol significantly increased around the time of the first egg. These findings are in accordance with other studies that also reported an increase of 17-β-estradiol plasma concentration before the onset of lay (Habig et al., 2021b; Mehlhorn et al., 2022).

After the initial increase at onset of lay, no significant changes within a genotype were observed. Thus, we could neither confirm a significant increase throughout the laying period (Habig et al., 2021b) nor a strong decrease in 17-β-estradiol between the 50th and 72nd week of age (Mehlhorn et al., 2022). Furthermore, there were no significant differences between the genotypes from the 33rd week of age onward when including F+ and F- animals. For both, the lack of significant differences could be related to the relatively high variation within the groups. Within the F+ animals, 17-β-estradiol was significantly higher in LSL than in JF and tended to be higher in Su than in JF. Thus, in contrast to our expectations and the findings of Mehlhorn et al. (2022), the plasma levels of 17-β-estradiol did not clearly reflect differences in laying performance between the genotypes. Even though 17-β-estradiol plasma levels were high in LSL, Su, that had a much lower laying performance, showed comparably high values, while JF, that have a similarly low laying performance as Su, had clearly lower 17-β-estradiol levels. Regarding these results, it should be taken into account that from the 35th/37th week of age the photoperiod was determined by the natural day length and that the genotypes in this study may have differed in their endocrinological response to it, as the response to changes in day length was affected by domestication (Karlsson et al., 2016).

When looking at the results for 17-β-estradiol and total calcium, it appears that both follow comparable patterns, e.g., higher values in follicle status positive LSL hens than in F + JF hens. This could be due to the influence of estrogen on the calcium metabolism. As Grunder et al. (1980b) observed, exogenous estradiol increases serum calcium and vitellogenin, a calcium-binding protein. The height of response, however, differed between genotypes.

The absolute values for estradiol plasma concentration obtained in this study should be viewed with caution, as samples had to be diluted, which could lead to deviating measurements and inaccuracy, especially in the lower concentration range.

### 4.7 Potential links between physiological parameters and keel bone fractures

Although the differences were not statistically significant, our results suggest that high-performing LSL are more susceptible to keel bone fractures than the low-performing chicken genotypes JF and Su, as fractures were only found in LSL. In order to identify potential internal factors that contribute to keel bone fractures, attention should be directed to traits, beyond laying performance, in which high-performing layers differ from low-performing genotypes.

Our results show that besides laying more eggs, LSL also have a higher eggshell weight and thickness and therefore a higher calcium output per egg than JF and Su. This could mean that eggshell formation requires more calcium from bone in this genotype and could promote demineralization of the skeleton over time. Within a layer line, other studies did not find a clear negative correlation between eggshell mass and bone quality (Jansen et al., 2020; Alfonso-Carrillo et al., 2021). However, the differences in eggshell mass in these studies were smaller than those between the high- and low-performing genotypes in our study.

Another factor is keel bone ossification in relation to onset of lay (Thøfner et al., 2021; Toscano et al., 2020). In LSL, a first egg was found 6 weeks earlier than in JF. However, both reached complete ossification of the keel bone at approximately the same age with approximately 75% of hens having completely ossified keel bones in the 25th week of age. Although the exact extent of keel bone ossification at the time is unknown, this suggests that ossification had further progressed in JF than in LSL at onset of lay. Additionally, radiographic density was not only lower in LSL than in Su in the 16th week of age, but also increased comparably across all three genotypes during the first half of the experiment. Taken together with their earlier onset of lay, this suggests that even the ossified part of the keel bone may be less mineralized at onset of lay in LSL compared to JF and Su. This would mean that in LSL, a major part of the ossification and mineralization of the keel bone takes place while the body already has to provide large amounts of calcium for the formation of the eggshell. Also, as our results indicate, plasma levels of reproductive hormones, especially 17-β-estradiol, are already higher and could therefore affect bone metabolism. In humans, estrogens play an important role in bone growth and maturation (as reviewed by Chargin and Sävendahl, 2006). However, relatively little is known about its impact on bone maturation and ossification in birds. In accordance with other studies (Dunn et al., 2021; Thøfner et al., 2021), our study points out that the early onset of lay and the early laying period play an important role in the pathogenesis of keel bone fractures.

## 5 Conclusion

Our study gives an insight into keel bone health and possible relationships to egg quality, bone, and blood parameters in two chicken genotypes that have not or not intensively been selected for laying performance. The results suggest that further research should investigate the role of keel bone mineralization and ossification at onset of lay in the etiology of keel bone fractures and the possibilities for breeding and management adaptations.

## Data Availability

The datasets presented in this study can be found in online repositories. The names of the repository/repositories and accession number(s) can be found below: 10.5281/zenodo.18127724.

## Supplementary material

SUPPLEMENTARY Table S1 LSM: body weight, keel bone, and blood parameters.

## References

[B1] Adler C. A. B. Shynkaruk T. McPhee S. Buchynski K. Herr A. Herwig E. (2024). Balancing act: studying the effect of perch space allowance on welfare in Canadian laying strain pullets raised in floor pens with access to a single-tier perch system to 18 wk of age. Poult. Sci. 103 (12), 104457. 10.1016/j.psj.2024.104457 39504835 PMC11570713

[B2] Ajala E. O. Eletta O. Ajala M. A. Oyeniyi S. K. (2018). Characterization and evaluation of chicken eggshell for use as a bio-resource. Arid Zone J. Eng. Technol. Environ. 14 (1), 26–40.

[B3] Alfonso-Carrillo C. Benavides-Reyes C. Los Mozos J. Dominguez-Gasca N. Sanchez-Rodríguez E. Garcia-Ruiz A. I. (2021). Relationship between bone quality, egg production and eggshell quality in laying hens at the end of an extended production cycle (105 weeks). Anim. (Basel) 11 (3), 623. 10.3390/ani11030623 PMC7996911 33652961

[B4] Armstrong E. A. Rufener C. Toscano M. J. Eastham J. E. Guy J. H. Sandilands V. (2020). Keel bone fractures induce a depressive-like state in laying hens. Sci. Rep. 10 (1), 3007. 10.1038/s41598-020-59940-1 32080271 PMC7033198

[B5] Aysel Ö. A. (1997). The variations in blood ionized calcium, sodium and potassium concentrations with age and laying cycle and the relationships of these ions with eggshell quality. Eur. Poult. Sci. 61 (6), 287–290. 10.1016/s0003-9098(25)01295-0

[B6] Buckner G. D. Insko W. M. Henry A. H. Wachs E. F. (1949). Rate of growth and calcification of the sternum of Male and female New Hampshire chickens having crooked keels. Poult. Sci. 28 (2), 289–292. 10.3382/ps.0280289

[B7] Baur S. Rufener C. Toscano M. J. Geissbühler U. (2020). Radiographic evaluation of keel bone damage in laying hens-morphologic and temporal observations in a longitudinal study. Front. Vet. Sci. 7, 129. 10.3389/fvets.2020.00129 32226794 PMC7081720

[B8] Bloom M. A. Domm L. V. Nalbandov A. V. Bloom W. (1958). Medullary bone of laying chickens. Am. J. Anatomie 102 (3), 411–453. 10.1002/aja.1001020304 13617222

[B9] Buckner G. D. Insko W. M. Henry A. H. Wachs E. F. (1948). Rate of growth and calcification of the sternum of Male and female New Hampshire chickens. Poult. Sci. 27 (4), 430–433. 10.3382/ps.0270430

[B10] Candelotto L. Stratmann A. Gebhardt-Henrich S. G. Rufener C. van de Braak T. Toscano M. J. (2017). Susceptibility to keel bone fractures in laying hens and the role of genetic variation. Poult. Sci. 96 (10), 3517–3528. 10.3382/ps/pex146 28938772

[B11] Casey-Trott T. Heerkens J. L. T. Petrik M. Regmi P. Schrader L. Toscano M. J. (2015). Methods for assessment of keel bone damage in poultry. Poult. Sci. 94 (10), 2339–2350. 10.3382/ps/pev223 26287001

[B12] Casey-Trott T. M. Korver D. R. Guerin M. T. Sandilands V. Torrey S. Widowski T. M. (2017). Opportunities for exercise during pullet rearing, part I: effect on the musculoskeletal characteristics of pullets. Poult. Sci. 96 (8), 2509–2517. 10.3382/ps/pex059 28379533 PMC5850348

[B13] Chargin A. S. Sävendahl L. (2006). Estrogens and growth: review. Pediatr. Endocrinol. Rev. 4 (4), 329–334. 17643080

[B14] Dascălu R. Sabău M. Proteasa A. Schuszler L. Sala A. Șerb M. (2013). Contributions to the treatment of traumatic orthopedic disorders in birds. Scientific works. Series C. Veterinary Med. 59 (3), 77–84.

[B15] Donkó T. Tischler A. Csóka Á. Kovács G. Emri M. Petneházy Ö. (2018). Estimation of bone mineral density and breaking strength of laying hens based on scans of computed tomography for body composition analysis. Br. Poult. Sci. 59 (4), 365–370. 10.1080/00071668.2018.1471662 29786455

[B16] Dunn I. C. Fleming R. H. McCormack H. A. Morrice D. Burt D. W. Preisinger R. (2007). A QTL for osteoporosis detected in an F2 population derived from white leghorn chicken lines divergently selected for bone index. Anim. Genet. 38 (1), 45–49. 10.1111/j.1365-2052.2006.01547.x 17257187

[B17] Dunn I. C. Koning D.-J. de McCormack H. A. Fleming R. H. Wilson P. W. Andersson B. (2021). No evidence that selection for egg production persistency causes loss of bone quality in laying hens. Genet. Sel. Evol. 53 (1), 11. 10.1186/s12711-021-00603-8 33541269 PMC7860618

[B18] Endo H. Tsunekawa N. Kudo K. Oshida T. Motokawa M. Sonoe M. (2022). Comparative morphological study of skeletal muscle weight among the red jungle fowl (gallus gallus) and various fowl breeds (*Gallus domesticus*). J. Exp. Zool. B Mol. Dev. Evol. 338 (8), 542–551. 10.1002/jez.b.23111 34826346 PMC9788176

[B19] Eusemann B. K. Baulain U. Schrader L. Thöne-Reineke C. Patt A. Petow S. (2018a). Radiographic examination of keel bone damage in living laying hens of different strains kept in two housing systems. PLoS One 13 (5), 1–17. 10.1371/journal.pone.0194974 29742164 PMC5942800

[B20] Eusemann B. K. Sharifi A. R. Patt A. Reinhard A. K. Schrader L. Thöne-Reineke C. (2018b). Influence of a sustained release deslorelin acetate implant on reproductive physiology and associated traits in laying hens. Front. Physiol. 9, 1846. 10.3389/fphys.2018.01846 30618846 PMC6306558

[B21] Eusemann B. K. Patt A. Schrader L. Weigend S. Thöne-Reineke C. Petow S. (2020). The role of egg production in the etiology of keel bone damage in laying hens. Front. Vet. Sci. 7, 81. 10.3389/fvets.2020.00081 32154276 PMC7047165

[B22] Farmer M. Roland D. A. Clark A. J. (1986). Influence of dietary calcium on bone calcium utilization. Poult. Sci. 65 (2), 337–344. 10.3382/ps.0650337 3703779

[B23] Fawcett D. L. Casey-Trott T. M. Jensen L. Caston L. J. Widowski T. M. (2020). Strain differences and effects of different stocking densities during rearing on the musculoskeletal development of pullets. Poult. Sci. 99 (9), 4153–4161. 10.1016/j.psj.2020.05.046 32867958 PMC7598119

[B24] Fleming R. H. Korver D. McCormack H. A. Whitehead C. C. (2004). Assessing bone mineral density *in vivo:* digitized fluoroscopy and ultrasound. Poult. Sci. 83 (2), 207–214. 10.1093/ps/83.2.207 14979571

[B25] Garant R. Tobalske B. W. Sassi N. B. van Staaveren N. Widowski T. Powers D. R. (2022). Wing-feather loss in white-feathered laying hens decreases pectoralis thickness but does not increase risk of keel bone fracture. R. Soc. Open Sci. 9 (6), 220155. 10.1098/rsos.220155 35719889 PMC9198519

[B26] Gebhardt-Henrich S. G. Fröhlich E. K. F. (2015). Early onset of laying and bumblefoot favor keel bone fractures. Animals 5 (4), 1192–1206. 10.3390/ani5040406 26633520 PMC4693210

[B27] Gretarsson P. Søvik Å. Thøfner I. Moe R. O. Toftaker I. Kittelsen K. (2024). Fracture morphology and ossification process of the keel bone in modern laying hens based on radiographic imaging. PLoS One 19 (10), e0312878. 10.1371/journal.pone.0312878 39471193 PMC11521247

[B28] Grunder A. A. Guyer R. B. Buss E. G. Clagett C. O. (1980a). Calcium-binding proteins in serum: quantitative differences between thick and thin shell lines of chickens. Poult. Sci. 59 (4), 880–884. 10.3382/ps.0590880 7375434

[B29] Grunder A. A. Guyer R. B. Buss E. G. Clagett C. O. (1980b). Effect of estradiol on calcium and calcium binding in serum of thick and then-shell lines of chickens. Poult. Sci. 59 (12), 2776–2781. 10.3382/ps.0592776 7267524

[B30] Habig C. Henning M. Baulain U. Jansen S. Scholz A. M. Weigend S. (2021a). Keel bone damage in laying hens-its relation to bone mineral density, body growth rate and laying performance. Animals 11 (6), 1546. 10.3390/ani11061546 34070496 PMC8228274

[B31] Habig C. Weigend A. Baulain U. Petow S. Weigend S. (2021b). Influence of age and phylogenetic background on blood parameters associated with bone metabolism in laying hens. Front. Physiol. 12, 678054. 10.3389/fphys.2021.678054 33995131 PMC8117343

[B32] Hanlon C. Takeshima K. Kiarie E. G. Bédécarrats G. Y. (2022). Bone and eggshell quality throughout an extended laying cycle in three strains of layers spanning 50 years of selection. Poult. Sci. 101 (3), 101672. 10.1016/j.psj.2021.101672 35074590 PMC8789532

[B33] Hildebrand L. Gerloff C. Winkler B. Eusemann B. K. Kemper N. Petow S. (2024). Japanese quails (Coturnix japonica) show keel bone damage during the laying period-a radiography study. Front. Physiol. 15, 1368382. 10.3389/fphys.2024.1368382 38545371 PMC10967949

[B34] Hiyama S. Sugiyama T. Kusuhara S. Uchida T. (2012). Evidence for estrogen receptor expression during medullary bone formation and resorption in estrogen-treated Male Japanese quails (coturnix Coturnix japonica). J. Vet. Sci. 13 (3), 223–227. 10.4142/jvs.2012.13.3.223 23000578 PMC3467396

[B35] Hocking P. M. Bain M. Channing C. E. Fleming R. Wilson S. (2003). Genetic variation for egg production, egg quality and bone strength in selected and traditional breeds of laying fowl. Br. Poult. Sci. 44 (3), 365–373. 10.1080/0007166031000085535 12964619

[B36] Jain K. Panigrahi M. Nayak S. S. Rajawat D. Sharma A. Sahoo S. P. (2024). The evolution of contemporary livestock species: insights from mitochondrial genome. Gene 927, 148728. 10.1016/j.gene.2024.148728 38944163

[B37] Jansen S. Baulain U. Habig C. Weigend A. Halle I. Scholz A. M. (2020). Relationship between bone stability and egg production in genetically divergent chicken layer lines. Animals (Basel) 10 (5), 850. 10.3390/ani10050850 32423072 PMC7278460

[B38] Jung L. Niebuhr K. Hinrichsen L. K. Gunnarsson S. Brenninkmeyer C. Bestman M. (2019). Possible risk factors for keel bone damage in organic laying hens. Animal 13 (10), 2356–2364. 10.1017/S175173111900003X 30808429

[B39] Jung L. Hillemacher S. Tiemann I. Lepke M. Hinrichs D. (2024). Presence of keel bone damage in laying hens, pullets and roosters of local chicken breeds. PLoS One 19 (1), e0297586. 10.1371/journal.pone.0297586 38277352 PMC10817119

[B40] Käppeli S. Gebhardt-Henrich S. G. Fröhlich E. Pfulg A. Stoffel M. H. (2011). Prevalence of keel bone deformities in Swiss laying hens. Br. Poult. Sci. 52 (5), 531–536. 10.1080/00071668.2011.615059 22029778

[B41] Karlsson A.-C. Fallahshahroudi A. Johnsen H. Hagenblad J. Wright D. Andersson L. (2016). A domestication related mutation in the thyroid stimulating hormone receptor gene (TSHR) modulates photoperiodic response and reproduction in chickens. Gen. Comp. Endocrinol. 228, 69–78. 10.1016/j.ygcen.2016.02.010 26873630

[B42] Kittelsen K. E. Gretarsson P. Jensen P. Christensen J. P. Toftaker I. Moe R. O. (2021). Keel bone fractures are more prevalent in white leghorn hens than in red jungle fowl hens-A pilot study. PLoS One 16 (7), e0255234. 10.1371/journal.pone.0255234 34314465 PMC8315525

[B43] Kolakshyapati M. Flavel R. J. Sibanda T. Z. Schneider D. Welch M. C. Ruhnke I. (2019). Various bone parameters are positively correlated with hen body weight while range access has no beneficial effect on tibia health of free-range layers. Poult. Sci. 98 (12), 6241–6250. 10.3382/ps/pez487 31504903 PMC8913749

[B44] Lordelo M. Cid J. Cordovil CMDS Alves S. P. Bessa R. J. B. Carolino I. (2020). A comparison between the quality of eggs from Indigenous chicken breeds and that from commercial layers. Poult. Sci. 99 (3), 1768–1776. 10.1016/j.psj.2019.11.023 32111337 PMC7587768

[B45] Mans C. Taylor W. M. (2008). Update on neuroendocrine regulation and medical intervention of reproduction in birds. Vet. Clin. North Am. Exot. Anim. Pract. 11 (1), 83–105. vi. 10.1016/j.cvex.2007.09.003 18165139

[B46] Mehlhorn J. Höhne A. Baulain U. Schrader L. Weigend S. Petow S. (2022). Estradiol-17ß is influenced by age, housing system, and laying performance in genetically divergent laying hens (gallus gallus f.d.). Front. Physiol. 13, 954399. 10.3389/fphys.2022.954399 35936910 PMC9353941

[B47] Mueller W. J. Schraer R. Schraer H. (1964). Calcium metabolism and skeletal dynamics of laying pullets. J. Nutr. 84, 20–26. 10.1093/jn/84.1.20 14210016

[B48] Nasr M. A. F. Nicol C. J. Murrell J. (2012). Do laying hens with keel bone fractures experience pain? PLoS One 7 (8), e42420. 10.1371/journal.pone.0042420 22927930 PMC3425496

[B49] Nasr M. A. F. Murrell J. Nicol C. J. (2013). The effect of keel fractures on egg production, feed and water consumption in individual laying hens. Br. Poult. Sci. 54 (2), 165–170. 10.1080/00071668.2013.767437 23647178

[B50] Nelson J. R. Settar P. Berger E. Wolc A. O’Sullivan N. Archer G. S. (2020). Brown and white egg-layer strain differences in fearfulness and stress measures. Appl. Animal Behav. Sci. 231, 105087. 10.1016/j.applanim.2020.105087

[B51] Nielsen S. Alvarez J. Bicout D. Calistri P. Canali E. Drewe J. (2023). Welfare of laying hens on farm. Efsa J. 21 (2), 7789. 10.2903/j.efsa.2023.7789 PMC9941850 36824680

[B52] North M. O. (1939). Breastbones of turkeys in relation to roosting. Univ. Wyo. Agric. Exp. Stn. Bull. 232, 3–12.

[B53] Parsons A. H. Combs G. F. (1981). Blood ionized calcium cycles in the chicken. Poult. Sci. 60 (7), 1520–1524. 10.3382/ps.0601520 7198779

[B54] Pighi L. Salvagno G. L. Ferraro R. Celegon G. Henry B. M. Lippi G. (2024). Impact of an air bubble within the syringe on test results obtained with a modern blood gas analyzer. J. Med. Biochem. 43 (5), 690–695. 10.5937/jomb0-49870 39712506 PMC11662954

[B55] Preisinger R. (2018). Innovative layer genetics to handle global challenges in egg production. Br. Poult. Sci. 59 (1), 1–6. 10.1080/00071668.2018.1401828 29129115

[B56] Pulcini D. Mattioli S. Angelucci E. Chenggang W. Cartoni Mancinelli A. Napolitano R. (2023). Shape and fractures of carina sterni in chicken genotypes with different egg deposition rates reared indoor or free-range. Sci. Rep. 13 (1), 22495. 10.1038/s41598-023-49909-1 38110659 PMC10728074

[B57] Qasir H. Reyer H. Oster M. Ponsuksili S. Trakooljul N. Sommerfeld V. (2025). Effects of a transient lack of dietary mineral phosphorus on renal gene expression and plasma metabolites in two high-yielding laying hen strains. BMC Genomics 26 (1), 129. 10.1186/s12864-025-11294-6 39930376 PMC11812262

[B58] Qin H. Jiao W. (2022). Correlation of muscle mass and bone mineral density in the NHANES US general population, 2017-2018. Med. Baltim. 101 (39), e30735. 10.1097/MD.0000000000030735 36181112 PMC9524880

[B59] Rassetafeln huehner (2024). Rassetafeln huehner. Available online at: https://www.bdrg.de/media/docs/Rassetafeln_Huehner.pdf (Accessed November 22, 2025).

[B60] Rentsch A. K. Ross E. Harlander A. Niel L. Siegford J. M. Widowski T. M. (2023). The development of laying hen locomotion in 3D space is affected by early environmental complexity and genetic strain. Sci. Rep. 13 (1), 10084. 10.1038/s41598-023-35956-1 37344513 PMC10284819

[B61] Riber A. B. Casey-Trott T. M. Herskin M. S. (2018). The influence of keel bone damage on welfare of laying hens. Front. Vet. Sci. 5, 6. 10.3389/fvets.2018.00006 29541640 PMC5835507

[B62] Roberts J. R. (2004). Factors affecting egg internal quality and egg shell quality in laying hens. J. Poult. Sci. 41 (3), 161–177. 10.2141/jpsa.41.161

[B63] Rodbard D. (1974). Statistical quality control and routine data processing for radioimmunoassays and immunoradiometric assays. Clin. Chem. 20 (10), 1255–1270. 4370388

[B64] Romanov M. N. Weigend S. (2001). Analysis of genetic relationships between various populations of domestic and jungle fowl using microsatellite markers. Poult. Sci. 80 (8), 1057–1063. 10.1093/ps/80.8.1057 11495455

[B65] Sadiq N. M. Anastasopoulou C. Patel G. Badireddy M. (2025). StatPearls: hypercalcemia. Treasure Island (FL). 28613465

[B66] Schmidt H. (1999). Hühner und Zwerghühner: handbuch Rasse-und Ziergeflügel. 2nd ed. Stuttgart: Ulmer.

[B67] Schütz K. Kerje S. Carlborg O. Jacobsson L. Andersson L. Jensen P. (2002). QTL analysis of a red junglefowl × white leghorn intercross reveals trade-off in resource allocation between behavior and production traits. Behavor Genet. 32 (6), 423–433. 10.1023/a:1020880211144 12467340

[B68] Sheen J. R. Mabrouk A. Garla V. V. (2025). StatPearls: fracture healing overview. Treasure Island (FL). 31869142

[B69] Thiele H.-H. (2012). Management tools to influence egg weight in commercial layers. Lohmann Inf. 47, 21–31.

[B70] Thøfner I. Hougen H. P. Villa C. Lynnerup N. Christensen J. P. (2020). Pathological characterization of keel bone fractures in laying hens does not support external trauma as the underlying cause. PLoS One 15 (3), e0229735. 10.1371/journal.pone.0229735 32150551 PMC7062247

[B71] Thøfner I. C. N. Dahl J. Christensen J. P. (2021). Keel bone fractures in Danish laying hens: prevalence and risk factors. PLoS One 16 (8), e0256105. 10.1371/journal.pone.0256105 34388183 PMC8362975

[B72] Toscano M. Booth F. Richards G. Brown S. Karcher D. Tarlton J. (2018). Modeling collisions in laying hens as a tool to identify causative factors for keel bone fractures and means to reduce their occurrence and severity. PLoS One 13 (7), e0200025. 10.1371/journal.pone.0200025 29990363 PMC6038993

[B73] Toscano M. J. Dunn I. C. Christensen J.-P. Petow S. Kittelsen K. Ulrich R. (2020). Explanations for keel bone fractures in laying hens: are there explanations in addition to elevated egg production? Poult. Sci. 99 (9), 4183–4194. 10.1016/j.psj.2020.05.035 32867962 PMC7597989

[B74] Tracy L. M. Temple S. M. Bennett D. C. Sprayberry K. A. Makagon M. M. Blatchford R. A. (2019). The reliability and accuracy of palpation, radiography, and sonography for the detection of keel bone damage. Animals 9 (11), 894. 10.3390/ani9110894 31683826 PMC6912489

[B75] Wang S. McDonnell E. H. Sedor F. A. Toffaletti J. G. (2002). pH effects on measurements of ionized calcium and ionized magnesium in blood. Arch. Pathol. Lab. Med. 126 (8), 947–950. 10.1043/0003-9985(2002)126<0947:PEOMOI>2.0.CO;2 12171493

[B76] Warren D. C. (1937). Physiologic and genetic studies of crooked keels in chicken. Kans. Tech. Bull. 44, 2–32.

[B77] Wei H. Chen Y. Nian H. Wang J. Liu Y. Wang J. (2021). Abnormal bone metabolism may be a primary causative factor of keel bone fractures in laying hens. Animals 11 (11), 3133. 10.3390/ani11113133 34827866 PMC8614394

[B78] Whitehead C. C. (1992). Bone biology and skeletal disorders in poultry: no. 23 (poultry science symposium S.) (Oxfordshire: Carfax Pub. Co.).

[B79] Whitehead C. C. Fleming R. H. (2000). Osteoporosis in cage layers. Poult. Sci. 79 (7), 1033–1041. 10.1093/ps/79.7.1033 10901207

[B80] Wilkins L. J. McKinstry J. L. Avery N. C. Knowles T. G. Brown S. N. Tarlton J. (2011). Influence of housing system and design on bone strength and keel bone fractures in laying hens. Vet. Rec. 169 (16), 414. 10.1136/vr.d4831 21862469

[B81] Wilson S. Thorp B. H. (1998). Estrogen and cancellous bone loss in the fowl. Calcif. Tissue Int. 62 (6), 506–511. 10.1007/s002239900470 9576978

