# Supplementary material for: Comparison of keel bone traits, eggshell production, and physiological parameters between a hybrid layer and two low-performing chicken genotypes
Source: Front Physiol. 2026 Mar 13;17:1782139. doi: 10.3389/fphys.2026.1782139 (PMC13021905; doi:10.3389/fphys.2026.1782139)
Supplement: Supplementary file 1 [file Table1.docx]

| **Comparison** | **Body weight [g]** | **Keel Bone Length [cm]** | **Radiographic Density [mmAleq]** | **POD [%]**  **(16^th^ week of age excluded)** | **Pectoral Muscle Thickness [cm]** | **Calcium [mmol/l]** | **Phosphate [mmol/l]** | **iCa [mmol/l]** | **Estradiol [pg/ml]** |
| --- | --- | --- | --- | --- | --- | --- | --- | --- | --- |
| **Genotype** |  |  |  |  |  |  |  |  |  |
| JF |  |  |  | 0.9±0.26 ^b^ | 1.67±0.04 ^a^ |  |  |  |  |
| LSL |  |  |  | 0.52±0.21 ^ab^ | 1.77±0.05 ^a^ |  |  |  |  |
| Su |  |  |  | 0.17±0.12 ^a^ | 2.1±0.05 ^b^ |  |  |  |  |
|  |  |  |  |  |  |  |  |  |  |
| **Follicle Status x Genotype** | |  |  |  |  |  |  |  |  |
| F- x JF |  |  |  |  |  | 2.77±0.11 ^a^ | 1.01±0.07 ^a^ | 1.28±0.06 n.s. | 125.91±13.92 ^a^ |
| F- x LSL |  |  |  |  |  | 2.74±0.17 ^a^ | 1.79±0.1 ^c^ | 1.06±0.19 n.s. | 158.66±28.15 ^a^ |
| F- x Su |  |  |  |  |  | 2.93±0.11 ^a^ | 1.24±0.09 ^ab^ | 1.42±0.04 n.s. | 181.82±26.54 ^ab^ |
| F+ x JF |  |  |  |  |  | 3.57±0.19 ^b^ | 1.05±0.06 ^a^ | 1.32±0.06 n.s. | 296.54±24.17 ^bc^ |
| F+ x LSL |  |  |  |  |  | 5.66±0.22 ^c^ | 1.45±0.06 ^b^ | 1.44±0.04 n.s. | 429.47±37.36 ^d^ |
| F+ x Su |  |  |  |  |  | 4.48±0.37 ^bc^ | 1.21±0.18 ^abc^ | 1.54±0.09 n.s. | 493.36±75.96 ^cd^ |
|  |  |  |  |  |  |  |  |  |  |
| **Age x Genotype** |  |  |  |  |  |  |  |  |  |
| 16 x JF | 558.57±38.25 ^a^ | 8.4±0.15 ^a^ | 1.51±0.07 ^ab^ |  |  | 3±0.18 ^abc^ | 1.26±0.13 ^abcde^ |  | 65.42±8.02 ^ab^ |
| 16 x LSL | 1095.83±41.32 ^d^ | 10.83±0.16 ^c^ | 1.19±0.08 ^a^ |  |  | 2.6±0.07 ^ab^ | 1.89±0.07 ^f^ |  | 124.23±18.14 ^b^ |
| 16 x Su | 1133.75±41.32 ^d^ | 10.93±0.16 ^c^ | 1.84±0.08 ^bc^ |  |  | 2.65±0.03 ^ab^ | 1.83±0.08 ^f^ |  | 54.13±7.54 ^a^ |
| 25 x JF | 671.79±38.25 ^ab^ | 9.21±0.15 ^b^ | 2.28±0.07 ^def^ |  |  | 2.85±0.15 ^abc^ | 0.99±0.12 ^abc^ | 1.23±0.07 ^abc^ | 105.01±12.87 ^ab^ |
| 25 x LSL | 1518.33±41.32 ^e^ | 11.86±0.16 ^d^ | 2.2±0.08 ^cde^ |  |  | 5.04±0.28 ^ef^ | 1.26±0.11 ^bcde^ | 1.41±0.06 ^bc^ | 323.78±41.43 ^cd^ |
| 25 x Su | 1534.17±41.32 ^ef^ | 13.58±0.16 ^e^ | 2.03±0.08 ^cd^ |  |  | 2.53±0.04 ^a^ | 1.66±0.07 ^ef^ | 1.22±0.06 ^abc^ | 97.86±13.62 ^ab^ |
| 33 x JF | 832.5±38.25 ^c^ | 9.18±0.15 ^b^ | 2.64±0.07 ^g^ |  |  | 3.33±0.3 ^abcd^ | 0.99±0.07 ^ab^ | 1.47±0.06 ^cde^ | 272.57±34.69 ^c^ |
| 33 x LSL | 1795±41.32 ^gh^ | 12.23±0.16 ^d^ | 2.48±0.08 ^efg^ |  |  | 4.92±0.32 ^def^ | 1.27±0.08 ^bcd^ | 1.67±0.06 de | 400.39±51.23 ^cd^ |
| 33 x Su | 1786.25±41.32 ^g^ | 13.78±0.16 ^ef^ | 2.73±0.08 ^g^ |  |  | 3.09±0.32 ^abc^ | 0.85±0.12 ^ab^ | 1.46±0.06 ^cd^ | 370.99±49.41 ^cd^ |
| 50/52 x JF | 816.79±38.25 ^c^ | 9.37±0.15 ^b^ | 2.55±0.07 ^efg^ |  |  | 2.9±0.21 ^abc^ | 0.96±0.07 ^ab^ | 1.37±0.06 ^abcd^ | 297.69±39.4 ^cd^ |
| 50/52 x LSL | 1835±41.32 ^gh^ | 12.34±0.16 ^d^ | 2.66±0.08 ^fg^ |  |  | 6.04±0.26 ^f^ | 1.6±0.11 ^cdef^ | 1.49±0.06 ^cde^ | 519.48±69.15 ^cd^ |
| 50/52 x Su | 1967.13±42.26 ^h^ | 14.24±0.17 ^f^ | 2.7±0.08 ^g^ |  |  | 4.07±0.43 ^cdef^ | 1.03±0.17 ^abcd^ | 1.73±0.06 ^e^ | 583.56±77.78 ^d^ |
| 70/72 x JF | 785.74±40.7 ^bc^ | 9.36±0.16 ^b^ | 2.33±0.08 ^defg^ |  |  | 3.11±0.33 ^abc^ | 0.88±0.12 ^ab^ | 1.11±0.06 ^ab^ | 288.51±38.21 ^c^ |
| 70/72 x LSL | 1717.5±41.32 ^fg^ | 12.26±0.16 ^d^ | 2.47±0.08 ^efg^ |  |  | 6.04±0.53 ^ef^ | 1.65±0.13 ^def^ | 1.15±0.06 ^a^ | 476.7±60.99 ^cd^ |
| 70/72 x Su | 1859.85±42.26 ^gh^ | 14.11±0.17 ^ef^ | 2.57±0.08 ^efg^ |  |  | 3.69±0.39 ^bcde^ | 0.73±0.07 ^a^ | 1.4±0.06 ^abcd^ | 334.63±46.59 ^cd^ |
|  |  |  |  |  |  |  |  |  |  |

**Supplementary Material 1: Least squares means of continuous keel bone parameters, blood parameters and body weight.** Significant differences (Tukey-Kramer, p < 0.05) are indicated by different letters.
